# Process control and *in silico* modeling strategies for enabling high density culture of human pluripotent stem cells in stirred tank bioreactors

**DOI:** 10.1016/j.xpro.2021.100988

**Published:** 2021-12-09

**Authors:** Felix Manstein, Kevin Ullmann, Wiebke Triebert, Robert Zweigerdt

**Affiliations:** 1 Leibniz Research Laboratories for Biotechnology and Artificial Organs (LEBAO), Hannover, Germany; 2 REBIRTH Cluster of Excellence, Hannover Medical School, Carl-Neuberg-Str. 1, 30625 Hannover, Germany

**Keywords:** Biotechnology and bioengineering, Cell culture, Stem Cells

## Abstract

The routine therapeutic and industrial applications of human pluripotent stem cells (hPSCs) require their constant mass supply by robust, efficient, and economically viable bioprocesses. Our protocol describes the fully controlled expansion of hPSCs in stirred tank bioreactors (STBRs) enabling cell densities of 35 × 10^6^ cells/mL while reducing culture medium consumption by 75%. This is achieved by *in silico* process modeling and computable upscaling. We provide a detailed blueprint for systematic process development of hPSCs and their progenies.

For complete details on the use and execution of this protocol, please refer to Manstein et al. (2021).

## Highlights

• Optimized protocol for high-density expansion of pluripotent stem cells
• Guideline for adoption of the *in silico* model
• Universal upscaling strategy for aggregate-based processes in stirred bioreactors
• Blueprint for advancing process automation and lineage differentiation strategies

## Graphical abstract

**Figure:**
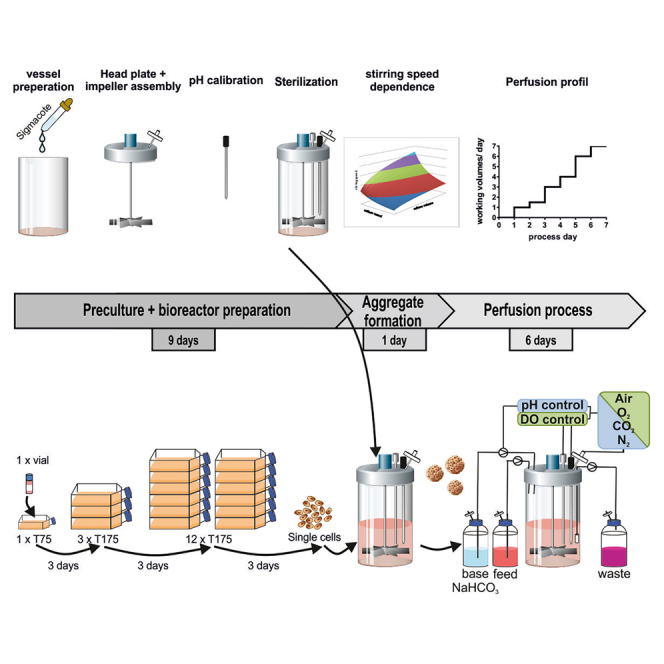

## Before you begin

Several human pluripotent stem cell (hPSC)-based therapy concepts have now progressed towards first-in-man studies (Kobold et al., 2020). However, leading investigators highlighted the current need for more efficient culture processes for generating pluripotent hPSCs - the “raw material” for lineage-directed differentiations - at clinically relevant quantities, quality and at commercially viable conditions (Ackermann et al., 2018; Stevens and Murry, 2018; Halloin et al., 2019; Sahabian et al., 2021).

In 2019, we published a basic bioreactor-based expansion protocol for hPSCs (Manstein et al., 2019). More recently, (Manstein et al., 2021) we could show the overarching importance of pH control for maintaining hPSC proliferation, in line with the established knowledge from biotechnology that the proliferation of continuous cell lines is diminished at pH levels below 7.0. Moreover, in contrast to continuous cell lines such as CHO and BHK cells, hPSCs do not consume lactate, which they extensively produce. This leads to the combinatorial challenge of rising glucose demands further boosting lactate secretion, consequently driving pH stabilizing base supplementation, together elevating osmolality levels. Given these closely interlinked effects of culture strategies and the cell metabolism, we found that perfusion feeding is the most appropriate approach for enabling the controlled high-density cultivation of hPSCs grown aggregates in suspension. Perfusion feeding allows for the proliferation- and density- specific adaption of the media perfusion rate, thus stabilizing culture conditions by providing fresh nutrients, while simultaneously diluting growth-inhibiting metabolites such as lactate. In addition, we demonstrate in this paper how the mean aggregate size and the aggregate size distribution can be effectively controlled by the stirring speed; this approach can be utilized for computable process upscaling and transition to larger bioreactor systems as well.

Considering the above outlined challenges, this protocol describes a strategy for hPSC bioprocessing enabling the 70-fold expansion of suspension-seeded cells within 7 days in stirred tank bioreactors (STBRs) ultimately yielding 35 × 10^6^ cells/mL. This expansion rate and cell density is achieved by combining the advantages of perfusion feeding, the tight control of critical process parameters such as pH and dissolved oxygen (DO) as well as uninterrupted nutrient supply facilitated by *in silico* process modelling. In addition, a universally applicable upscaling strategy is revealed, accounting for bioreactor system-dependent hardware features such as the vessel- and impeller- dimensions.

The proposed culture strategy is thoroughly validated with three independent hiPSC lines, strongly suggesting its universal applicability and similar efficiency for any hiPSC or hESC lines at hand and its potential utility for pluripotent stem cell lines from other species as well, which may also be of interest to the envisioned production of *in vitro* meat (Rubio et al., 2020).

For the successful protocol application, investigators should be generally trained in relevant mammalian cell culture techniques, including cryopreservation and thawing, sterile cell handling and passaging as well as established analytical standard techniques such as cell counting and viability staining.

Additional knowhow in bioreactor and process technologies such as: bioreactor assembly, bioreactor sterilization and handling under the sterile hood, suspension culture inoculation and process initiation/ execution as well as process monitoring and analysis.

### Background and guidance for applying the *in silico* model

Guided by our step-by-step protocol, investigators are enabled to recapitulate this advanced culture strategy and to achieve equivalent results using their specific hPSC line and respective STBR system at hand, without major adaptations. However, following this blueprint (model code can be found under Data S1), preexisting wet-lab data established in an individual laboratory can also be used to eventually draft and optimize their own, customized bioreactor- and cell-line specific *in silico* model.

The *in silico* model underlying our optimized process (Manstein et al., 2021), is based on classical Monod-kinetics (Monod, 1949) and considers:

1. key process factors that serve as so-called “Monod-variables”
  a. glucose-concentration
  b. lactate-concentration
  c. glutamine-concentration
  d. perfusion rate
  e. osmolality
  f. aggregate size
2. “Monod- (like-) constants, which serve as limiting constants for each respective variable.
3. cell-specific rates, which must be derived from existing process data, including:
  a. cell specific growth rate
  b. consumption rates for glucose and glutamine
  c. production rate for lactate
  d. cell line-dependent aggregate formation rate, which was found to be divers between individual hiPSC lines.

Potential starting values to establish such a model are displayed in Table 1.

**Table 1:** Exemplary kinetic model parameters

Model parameter	Value
K_Glc_ [mM]	1.5
K_Lac_ [mM]	50
K_Gln_ [mM]	0.01
K_Agg_ [μm]	350/2
K_Osm_ [mOsm/kg]	500
μ [d^−1^]	1.35
q_Glc_ [× 10^−8^ mmol × cell^−1^ × d^−1^]	1.474
q_Lac_ [× 10^−8^ mmol × cell^−1^ × d^−1^]	2.37
q_Gln_ [× 10^−9^ mmol × cell^−1^ × d^−1^]	1.856
agg_f_ [–]	0.95
agg_g_ [–]	0.25

**Figure 1:**
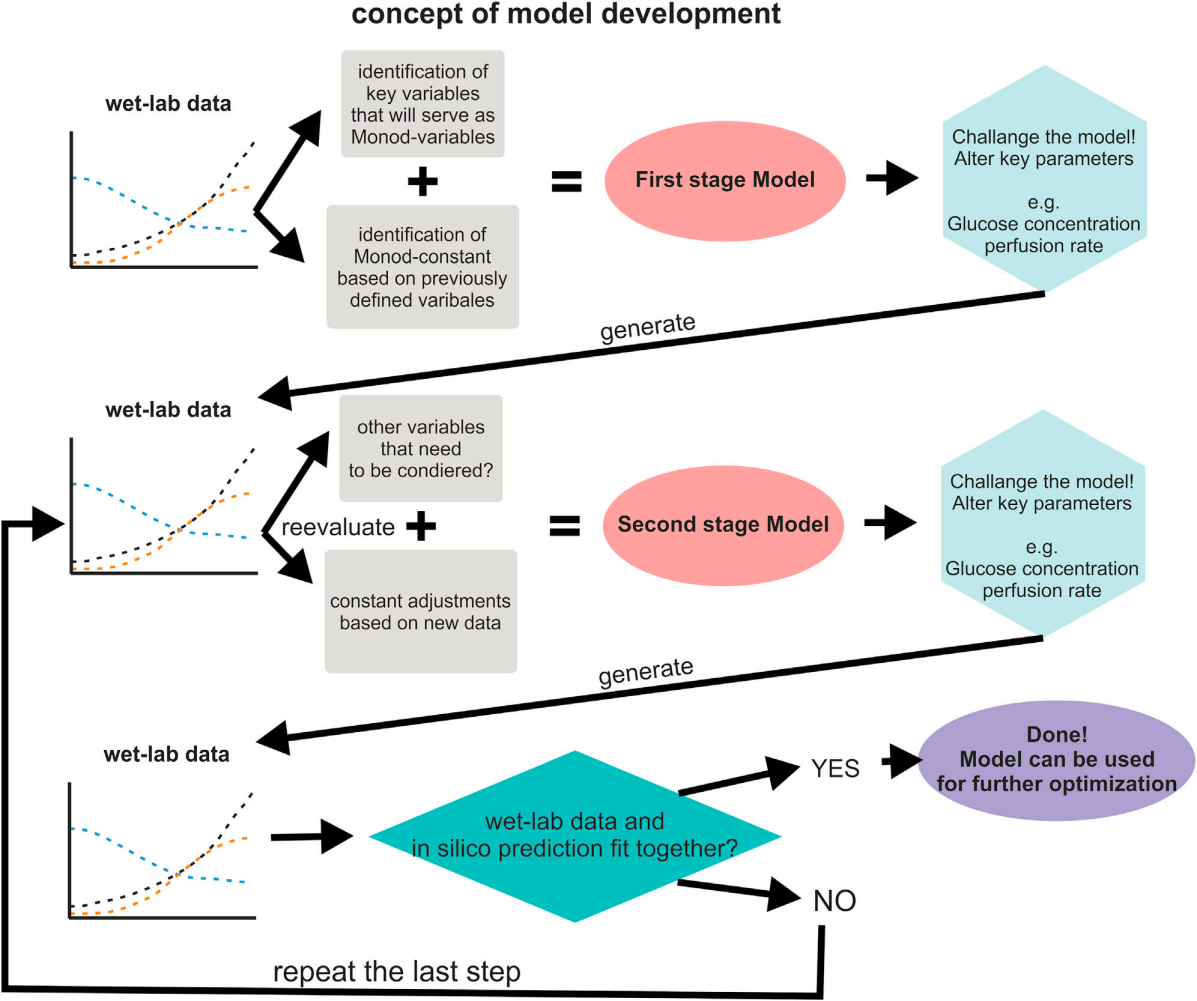
Schematic outline of the Monod-Model process development strategy In general, the process starts with already existing wet-lab data, which is an existing process that is supposed to be optimized. From this data, key variables which are regarded to majorly influence the process need to be identified. These variables are quite often metabolites like glucose, but also can be more complex like osmolality or even aggregate size (for aggregate dependent cultures). For these values so called “Monod-constants” need to be defined which are limiting values/ concentrations at which cell growth is impacted. Based on this the first stage model can be built. Afterwards this model needs to be challenged, first in silico and afterwards in the wet-lab. This means that key variables need to be altered in order to affect cell growth. Afterwards the impact of the alteration between the in silico model and the wet-lab need to be compared. With a high probability, the model does not align with the wet-lab data. In that case, the system needs to be reevaluated in order to identify new variables that need to be added, or to adjust the already existing monod-constants. Afterwards the second stage model is generated. From now on the steps of model alteration in silico and in the wet-lab followed by reevaluation need to be repeated until wet-lab and in silico data overlap.

The general workflow to establish/adjust the *in silico* model to a specific process is as follows (and displayed in Figure 1):

4. Establish a first stage model with potential starting values from Table 1 or the individual process
5. Challenge the model by applying alterations of key process parameters *in silico.*
6. Evaluation of the alterations *in situ* in the laboratory. The practical testing of the model will display its potential limitations.
7. Adaption the Monod- (like-) constants so that a second stage model better reflects the outcome of the *in situ* experiments. Potentially, the addition of new process-relevant factors must be considered as well, which may have been missing the first stage.
8. Repeat this model-challenging-and-adjustments” strategy until the *in silico* model and its predictions properly reflect the real wet lab results and process pattern.

To better illustrate the adjustment process the following description is given: For example, the Monod constant for lactate (K_Lac_) has been set to a relatively high level of 200 mM (rather than a more typical value of 50 mM). In consequence, the model may suggest the supplementation of high glucose concentration (for achieving high cell yields) in compliance with high lactate concentration, due to the high value defined for K_Lac_. Most likely, however, respective wet-lab experiments would result in poor cell proliferation in reply to detrimental lactate concentrations accumulating over time and thus inferior cell yields, but even without reaching the maximum lactate concentration level predicted by the model. As a result, the observed wet-lab data will serve as the basis for adapting the next stage model, reflecting reduction of K_Lac_; consequently, the new model should better reflect the cell growth and lactate concentration curves observed by the wet-lab experiments.

### Preparation of cultivation flasks for adherent culture

**Timing: 1.5 h**

9. Preparation of Geltrex-stocks and coating of T-flasks
  a. Preparation of aliquots from stock vials
    i. Thaw vial for 12 h on ice in the fridge at 4°C.
    ii. Aliquot à 125 μL and store at −80°C for up to 12 month.
  b. Coating of flasks
    i. Thaw an aliquot of 125 μL into 50 mL DMEM/F12 and mix.
    ii. 1 mL of Geltrex-DMEM/F12 mix is sufficient for 5 cm^2^ surface.
    iii. Incubate flasks in the incubator at 37°C for 1 h and store subsequently at 4°C for up to one month.
10. Alternative: Preparation of Vitronectin-stocks and coating of T-flasks
  a. Preparation of aliquots from stock vials
    i. Thaw vial at 20°C–22°C.
    ii. Aliquot à 250 μL and store at −80°C for up to 12 month.
  b. Coating of flasks
    i. Thaw an aliquot of 250 μL into 50 mL PBS and mix.
    ii. 1 mL of Vitronectin-PBS mix is sufficient for 10 cm^2^ surface.
    iii. Incubate flasks in the incubator at 37°C for 1 h and store subsequently at 4°C for up to one month.

## Key resources table

**Table:** 

REAGENT or RESOURCE	SOURCE	IDENTIFIER
**Antibodies**		

SSEA3 Monoclonal Antibody (MC-631), DyLight 650, Dilution 1:50	Thermo Fisher Scientific	Cat#MA1-020-D650
SSEA-4 Antibody, anti-human, VioBlue, REAfinity, Dilution 1:25	Miltenyi Biotec	Cat#130-098-366
TRA-1-60 Antibody, anti-human, PE, REAfinity, Dilution 1:25	Miltenyi Biotec	Cat#130-122-921
Oct3/4 Isoform A Antibody, anti-human/mouse, PE, REAfinity, Dilution 1:25	Miltenyi Biotec	Cat#130-123-771
Nanog Antibody, anti-human, APC, REAfinity, Dilution 1:25	Miltenyi Biotec	Cat#130-120-774
Ki-67 Antibody, anti-human/mouse, PE-Vio 770, REAfinity, Dilution 1:25	Miltenyi Biotec	Cat#130-120-419

**Chemicals, peptides, and recombinant proteins**		

PBS (10 ×), pH 7.4	Thermo Fisher Scientific	Cat#70011036
StemPro Accutase Cell Dissociation Reagent	Thermo Fisher Scientific	Cat#A1110501
Versene Solution	Thermo Fisher Scientific	Cat#15040033
DMEM/F-12, HEPES	Thermo Fisher Scientific	Cat#11330057
Pluronic F-68 Non-ionic Surfactant (100×)	Thermo Fisher Scientific	Cat#S6014
Geltrex LDEV-Free, hESC-Qualified, Reduced Growth Factor Basement Membrane Matrix	Thermo Fisher Scientific	Cat#A1413302
CTS Vitronectin (VTN-N) Recombinant Human Protein	Thermo Fisher Scientific	Cat#A27940
L-Glutamine 200 mM (100 ×)	Thermo Fisher Scientific	Cat#25030-024
D-(+)-Glucose Hybri-Max	Sigma-Aldrich	Cat#G5146
NaHCO_3_	Sigma-Aldrich	Cat#24040032
Na_2_SeO_3_	Sigma-Aldrich	Cat#S5261
Insulin solution human	Sigma-Aldrich	Cat#I9278
Bovine Serum Albumin	Sigma-Aldrich	Cat#A9418
Transferrin human recombinant	Sigma-Aldrich	Cat#T3705
Ascorbic acid 2-phosphate	Sigma-Aldrich	Cat#A8960
Sigmacote	Sigma-Aldrich	Cat#SL2
Recombinant Human TGF-β1	PeproTech	Cat#100-21C
Animal-Free Recombinant Human FGF-basic	PeproTech	Cat#AF-100-18B
y-27632 dihydrochloride	Tocris Bioscience	Cat#1254
FIX & PERM Kit	Dianova	Cat#GAS-002-1

**Experimental models: Cell lines**		

HSC1285_T-iPS2 (Hartung et al., 2013)	Hannover Medical School (MHH)	MHHi006-A
hHSC_Iso4_ADCF_SeViPS2 (Phönix) (Haase et al., 2017)	Hannover Medical School (MHH)	MHHi001-A
CD34+hPBHSC_GMPDU_SeV-iPS8 (Haase et al., 2019)	Hannover Medical School (MHH)	MHHi008-A

**Software and algorithms**		

FlowJo v10 software	BD Biosciences	N/A
Fiji ImageJ	N/A	N/A
DASware control	Eppendorf	Cat#78600167
Berkeley Madonna	University of California at Berkeley	N/A

**Other**		

10, 25 and 50 mL serological pipettes	Sarstedt	Cat#86.1254.001, Cat#86.1685.001, Cat#86.1689.001
15 and 50 mL conical tube	Greiner Bio-One	Cat#188261, Cat#227261
centrifuge tubes 500 mL	Corning Life Sciences	Cat#CLS431123-36EA
10 and 200 μL sterile filter pipette tips	Starlab	Cat#S1120-3810, Cat#S1120-8810
1000 μL sterile filter pipette tips	Sarstedt	Cat#70.762.211
T75 and T175 flasks	Greiner Bio-One	Cat#658175, Cat#660175
1.5 and 2.0 mL Eppendorf Safe-Lock Tubes	Eppendorf	Cat#0030120086, Cat#0030120094
500 mL bottle top filter	TPP	Cat#99505
Syringe filter	Carl Roth	Cat#P666.1
20 mL Syringe	B. Braun	Cat#4606205V
Cell culture cabinet/laminar flow (HERAsafe HS18)	Heraeus Instruments	N/A
Benchtop centrifuge (Heraeus Fresco 17)	Thermo Scientific	N/A
Benchtop centrifuge (Heraeus Multifuge 3 S-R)	Thermo Scientific	N/A
MACSQuant Analyzer 10 Flow Cytometer	Miltenyi Biotec	N/A
Vi-CELL XR	Beckman Coulter	N/A
Humidified incubator (MCO-20AIC)	Sanyo	N/A
Water bath	GFL	Cat#1003
Terg-a-zyme enzyme detergent pack	Sigma-Aldrich	Cat#Z273287-1EA
Disposable syringes, Omnifix 5 ml with Luer-lock	B. Braun	Cat#8728810F
Technical Buffer pH 4.01	WTW	Cat#108 800
Technical Buffer pH 7.00	WTW	Cat#108 802
Hamilton Storage Solution	Hamilton	Cat#238931
EasyFerm Bio PHI K8 120	Hamilton	Cat#243632-1513
OxyFerm FDA 225	Hamilton	Cat#237452
DASbox Mini bioreactor system for cell culture applications	Eppendorf	Cat#76DX04CC
DASbox Mini bioreactor vessel for cell culture applications	Eppendorf	Cat#76DS0250ODSS
DASbox exhaust system	Eppendorf	Cat#76DXOFF
DASbox exhaust condenser, Peltier	Eppendorf	Cat#76DXCOND
DASbox overhead drive	Eppendorf	Cat#76DXOHD
Pitched-Blade Impeller, 8-blade, 60° pitch, stainless steel, O.D. 34 mm, I.D. 5 mm	Eppendorf	Cat#78100604
Holding Sleeve, for 8-blade impeller, stainless steel with set screw, I.D. 8 mm, for shaft with O.D. 5 mm	Eppendorf	Cat#78100595
Compression Fitting, complete, with Pg 13.5 male thread, I.D. 12 mm	Eppendorf	Cat#78532284
Triple Port, Pg 13.5 thread, 3 tubes with O.D. 4 mm × L 85 mm, all parts included	Eppendorf	Cat#78706414
Pipe, stainless steel, with barb, O.D. 4 mm/I.D. 2 mm, L 225 mm	Eppendorf	Cat#78107023
Compression Fitting, complete, with Pg 13.5 male thread, I.D. 6 mm	Eppendorf	Cat#78532283
L-Sparger, stainless steel, complete, O.D. 6 mm, L 300 mm, W 63 mm	Eppendorf	Cat#77102022
Pump Head Tubing, for DASGIP MP8 pump, Bioprene, I.D. 0.5/W 1.05 mm, female/female	Eppendorf	Cat#78510118
Pump Head Tubing, for DASGIP MP8 pump, Bioprene, I.D. 1.0/W 1.05 mm, male/female	Eppendorf	Cat#78510109
Feed Line, with 2× Luer lock fittings, male/male, C-Flex, I.D. 0.8 mm, L 1 m	Eppendorf	Cat#78510309
Feed Line, with 2× Luer lock fittings, male/male, C-Flex, I.D. 0.8 mm, L 2 m	Eppendorf	Cat#78510310
Sampling Accessory, with swabable valve	Eppendorf	Cat#78510145
Polytetrafluoroethylene (PTFE) membrane inline vent filter; pore size 0.2 μm	mdi Membrane Technologies	Cat#ITFX0801BBXX109
Silicone tubing, inner diameter (i.d.) 1.0 mm, outer diameter (o.d.) 3.0 mm	Carl Roth	Cat#HC61.1
Silicone tubing, inner diameter (i.d.) 4.0 mm, outer diameter (o.d.) 6.0 mm	Carl Roth	Cat#HC65.2
hose reduction piece	Carl Roth	Cat#CT46.1
Female Luer-lock connector	Carl Roth	Cat#CT62.1
Male Luer-lock plug	Carl Roth	Cat#CT70.1
Connector, straight, female luer lock/tubing nipple, 4.8 mm	VWR	Cat#INFIPP-LFS48
Screw cap GL 45 with 2 hose connectors and EPDM gasket	Landgraf Laborsysteme (HLL)	Cat#102112807
Media bottle,250ml, two 9mm hose nozzles	Landgraf Laborsysteme (HLL)	Cat#L14040250
DASGIP Parallel Bioreactor System, for cell culture	Eppendorf	Cat#76DG04CCBB
DASGIP Vessel, DS1000ODSS, 350 mL – 1.0 L, 2× GL45 side arms	Eppendorf	Cat#76DS1000ODSS
Pitched-Blade Impeller, 8-blade, 60° pitch, stainless steel, O.D. 53 mm, I.D. 8 mm	Eppendorf	Cat#78100605
Holding Sleeve, for 8-blade impeller, stainless steel with set screw, I.D. 10 mm, for shaft with O.D. 8 mm	Eppendorf	Cat#78100597
Pump Head Tubing, for DASGIP® MP8 pump, Peripren, I.D. 2.0/W 0.8 mm, female/female	Eppendorf	Cat#78510237
EasyFerm Bio PHI K8 225	Hamilton	Cat#243632-1543
Media bottle,1000ml, two 9mm hose nozzles	Landgraf Laborsysteme (HLL)	Cat#L14040101
Sintered glass sparger, AD 6 × 15mm, Por.3, pore size 16–40 μm, glass tube AD 4 × 0.8 mm, L 35mm	Eppendorf	Cat#78903230
Stainless Steel Flange	Eppendorf	Cat#78107292

## Materials and equipment

• E8 basis medium (10 L)
  While working under the flow food, pour 20 bottles a 0.5 L of DMEM/F12 into a 10L glass bottle, while keeping the original bottles sterile.
  Add 5.43 g NaHCO_3_, adjust the pH to 7.4 with 5 M NaOH (should be 3–5 mL). Check the osmolality (should be 315–320 mOsm/kg).
  Filter to sterilize, aliquot a 500 mL in the old bottles and store at 4°C for up to 6 month.
• E8 feed basis medium I (10 L)
  Follow the instructions for E8 basis medium, and additionally add 30 g Glucose. Osmolality should be 335–340 mOsm/kg.
• E8 feed basis medium II (10 L)
  Follow the instructions for E8 basis medium, and additionally add 45 g Glucose. Osmolality should be 350–355 mOsm/kg.
• Rho-kinase inhibitor (RI) Y-27632 stocks (1000×, 10 mM)
  Dissolve 50 mg Y-27632 in 14.7 mL pure, filter to sterilize, aliquot a 1000 μL store at – 20°C for up to 12 month.
• bFGF stocks (1000×, 100 μg/mL)
  Add 300 μL of 10% Bovine serum albumin (BSA)-solution to 29.7 mL PBS to make a 0.1% BSA-solution.
  Dissolve 3000 μg recombinant bFGF (dehydrolyzed powder) in 30.0 mL of the 0.1 BSA-solution, filter to sterilize, aliquot a 1000 μL store at – 80°C for up to 12 month.
• TGF-β1 stocks (1000 ×, 2 μg/mL)
  Add 650 μL of 10% human albumin solution + 520 μL of 1 M HCl to 128.83 mL PBS to make a 0.05% human albumin/ 4 mM HCl solution and filter to sterilize.
  Dissolve 250 μg recombinant TGF-β1 (dehydrolyzed powder) in 125.0 mL of the prepared human albumin/HCl solution, aliquot a 1000 μL store at – 80°C for up to 12 month.
• Transferin stocks (1000 ×, 10.7 mg/mL)
  Dissolve 1.0 g recombinant Transferin (dehydrolyzed powder) in 93.46 mL PBS, filter to sterilize, aliquot a 1000 μL store at – 80°C for up to 12 month.
• L-ascorbic acid 2-phosphat stocks (1000 ×, 64 mg/mL)
  Dissolve 5.0 g L-ascorbic acid 2-phosphat in 78.13 mL PBS, filter to sterilize, aliquot a 1000 μL store at – 20°C.
• Sodium Selenite stocks (10000 ×, 140 μg/mL)
  Dissolve 0.014 g Sodium Selenite in 100 mL PBS, filter to sterilize, aliquot a 100 μL and store at – 20°C for up to 12 month.

**Table:** E8 adherent medium (for adherent (pre-) culture)

Reagent	Final concentration	Amount
E8 basis medium	N/A	500 mL
FGF2 (100 μg/mL)	100 μg/L	0.5 mL
TGF-β1 (2 μg/mL)	2 μg/L	0.5 mL
Insulin (10 mg/mL)	20 mg/L	1 mL
Ascorbic acid-2-phosphate (64 mg/mL)	64 mg/L	0.5 mL
Transferrin (10.7 mg/mL)	10.7 mg/L	0.5 mL
Sodium Selenite (140 μg/mL)	14 μg/L	0.05 mL
RI (Y-27632) (10 mM; only after passaging)	10 μM	0.5 mL
**Total**	**N/A**	**503.05 mL (503.55 mL)**

***Note:*** Stable under the following storage conditions: 4°C and maximum storage time of 2 weeks.

**Table:** E8 suspension medium (for bioreactor inoculation)

Reagent	Final concentration	Amount
E8 basis medium	N/A	500 mL
FGF2 (100 μg/mL)	100 μg/L	0.5 mL
TGF-β1 (2 μg/mL)	2 μg/L	0.5 mL
Insulin (10 mg/mL)	20 mg/L	1 mL
Ascorbic acid-2-phosphate (64 mg/mL)	64 mg/L	0.5 mL
Transferrin (10.7 mg/mL)	10.7 mg/L	0.5 mL
Sodium Selenite (140 μg/mL)	14 μg/L	0.05 mL
RI (Y-27632) (10 mM)	10 μM	0.5 mL
Pluronic™ F-68 Non-ionic Surfactant (10%)	0.1%	5 mL
**Total**	**N/A**	**508.55 mL**

***Note:*** Stable under the following storage conditions: 4°C and maximum storage time of 2 weeks.

**Table:** E8 full feed medium I (for perfusion feeding from day 1 – day 4)

Reagent	Final concentration	Amount
E8 basis feed medium I	N/A	500 mL
FGF2 (100 μg/mL)	100 μg/L	0.5 mL
TGF-β1 (2 μg/mL)	2 μg/L	0.5 mL
Insulin (10 mg/mL)	20 mg/L	1 mL
Ascorbic acid-2-phosphate (64 mg/mL)	64 mg/L	0.5 mL
Transferrin (10.7 mg/mL)	10.7 mg/L	0.5 mL
Sodium Selenite (140 μg/mL)	14 μg/L	0.05 mL
Pluronic™ F-68 Non-ionic Surfactant (10%)	0.1%	5 mL
L-Glutamine (200 mM)	+ 2 mM (4.5 mM in total)	5 mL
**Total**	**N/A**	**513.05 mL**

***Note:*** Stable under the following storage conditions: 4°C and maximum storage time of 2 weeks.

**Table:** E8 full feed medium II (for perfusion feeding from day 4 – day 7)

Reagent	Final concentration	Amount
E8 basis feed medium II	N/A	500 mL
FGF2 (100 μg/mL)	100 μg/L	0.5 mL
TGF-β1 (2 μg/mL)	2 μg/L	0.5 mL
Insulin (10 mg/mL)	20 mg/L	1 mL
Ascorbic acid-2-phosphate (64 mg/mL)	64 mg/L	0.5 mL
Transferrin (10.7 mg/mL)	10.7 mg/L	0.5 mL
Sodium Selenite (140 μg/mL)	14 μg/L	0.05 mL
Pluronic™ F-68 Non-ionic Surfactant (10%)	0.1%	5 mL
L-Glutamin (200 mM)	+ 2.5 mM (5.0 mM in total)	7.5 mL
**Total**	**N/A**	**515.55 mL**

***Note:*** Stable under the following storage conditions: 4°C and maximum storage time of 2 weeks.

## Step-by-step method details

### Thawing and adherent (pre-) culture of hPSCs for bioreactor inoculation

**Timing: 9 days**

The aim of this part of the protocol is to describe well-defined adherent (two dimensional, 2D) culture of hPSCs to generate the required quality and quantity of cells required for inoculating the suspension culture (3D) in bioreactors at the desired culture scale. The procedure described here details the generation of hPSCs sufficient to inoculate 4 × bioreactors at 150 mL scale, or alternatively 1 × bioreactor at 500 mL scale. When aiming at larger culture scales, one or two additional cell passages might be required to produce the respective cell numbers in 2D.

**Figure 2:**
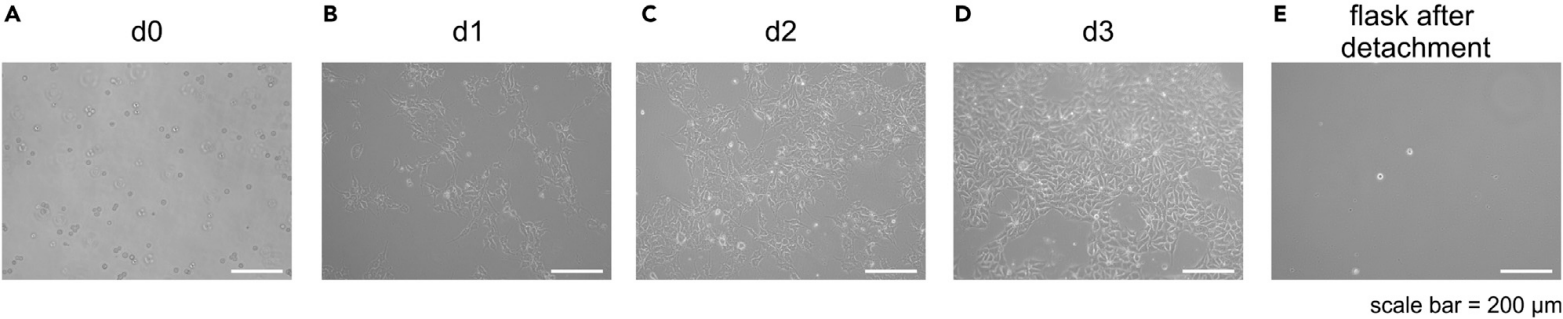
Exemplary microscopical pictures of Monolayer cell growth (A–E) Exemplary bright field images of a monolayer culture on Geltrex and in E8, directly after seeding (A), on day 1 (B), day 2 (C), day 3 (D) and of the flask after cell detachment in order to highlight good cell detachment (E) (scale bars = 200 µm).
  b. For passaging, wash cells with PBS, completely remove any residue of PBS and add 0.04 mL/cm^2^ StemPro™ Accutase™ and incubate for 3 min at 37°C. Check for cell detachment by tilting the flask.
  c. Dilute Accutase with the 3-fold volume of DMEM/F12, transfer into a conical tube and centrifuge at 300 × g for 3 min. Discard the supernatant, resuspend in 5 mL E8 adherent medium + 10 μM Y27632 and determine the cell count with the Vi-CELL XR (alternatively manually via Trypan blue exclusion and Neubauer chamber (Zweigerdt et al., 2011)).
  d. Remove the coating medium from three coated T175 flasks, add 35 mL E8 adherent medium + 10 μM Y27632 (0.2 mL/cm^2^) containing 3.5 × 10^6^ cells per flask (corresponding to 2 × 10^4^ cells/cm^2^).

***Note:*** If Vitronectin (instead of Geltrex) is used for the adherent culture, Versene might be used for cell detachment/passaging alternatively to Accutase; the procedure is identical to the steps above but the incubation time should be expanded to 10 min (as compared to 3 min suggested for Accutase treatment).

1. Thawing of cells
  a. Start with a high quality aliquot i.e., a vial of frozen hPSCs representing ∼3 × 10^6^ cells (previously generated e.g., by own adherent culture used for cryo-preservation).
  b. Thaw the vial at 37°C in a water bath until only a small rest of ice is visually observable.
  c. Spray the vial with ethanol and place it under a flow hood and dilute the suspension in 10-fold volume of DMEM/12 in a 15 mL conical tube.
  d. Centrifuge at 300 × g for 3 min. Discard the supernatant and resuspend in 15 mL E8 adherent medium + 10 μM Y27632. Remove the coating medium from one coated T75 flask and transfer the cell suspension into the flask.
  e. Place the T-flask in an incubator at 37°C and 5% CO_2_.
2. After 48 h of incubation, replace the old medium with 15 mL fresh E8 adherent medium and place the flask back in an incubator.
3. Passaging of cells
  a. 72 h after seeding (ideally not later), the cells should be passaged to ensure that cells remain at proliferative conditions optimal for this protocol; at this stage the culture confluence should be at ∼60–80% (exemplary pictures are shown in Figure 2).

4. After 48 h of incubation, replace the medium with 35 mL fresh E8 adherent medium per flask and place the flask back in an incubator.
5. 72 h after seeding, the cells should have reached ∼ 60–80% confluence and must be passaged to ensure optimal proliferative conditions for this protocol.
  a. For passaging, wash cells with PBS, completely remove any residue of PBS and add 0.04 mL/cm^2^ StemPro™ Accutase™ and incubate for 3 min at 37°C. Check and support cell detachment by tilting of the flask.
  b. Dilute Accutase with the 3-fold volume of DMEM/F12, transfer into a conical tube and centrifuge at 300 × g for 3 min. Discard the supernatant, resuspend in 25 mL E8 adherent medium + 10 μM Y27632 and determine the cell density with the Vi-CELL XR (alternatively manually via Trypan blue exclusion and Neubauer chamber).
  c. Remove the coating medium from twelve coated T175 flasks, add 35 mL E8 adherent medium + 10 μM Y27632 (0.2 mL/cm^2^), and 3.5 × 10^6^ cells per flask (2 × 10^4^ cells/cm^2^).
6. After 48 h of incubation, replace the old medium with 35 mL fresh E8 adherent medium per flask and place the flasks back in an incubator.
7. Observe the cells 72 h after seeding. The confluence should be ∼60%–80% and the medium should be slightly yellow and clear. For the inoculation of 4 × bioreactors at 150 mL scale, or 1 × bioreactor at 500 mL scale, the next major step “Bioreactor inoculation” should be followed; otherwise (to generate more cells for inoculating multiple bioreactors and/ or larger scale processes) repeat the steps 3–5, while respectively increased the number of T175 flasks.

**CRITICAL:** The seeding density into T-flasks might require slight (cell line-dependent) adjustment for your hPSC line at hand. However, it is highly critical that cells do not exceed the suggested ∼60%–80% confluence within the 72 h suggested for passaging; higher confluence and/ or exceeding the passaging time beyond 72 h may negatively impact on cell viability after bioreactor inoculation and subsequently reduce the process robustness, efficiency and overall protocol reproducibility. Moreover, even if the cells are not fully detached after 3 minutes of Accutase treatment, do not prolong this incubation step, to avoid detrimental effects on cell viability and proliferation; if the confluence and the passaging interval is following the above-described range, 3 min of Accutase treatment is resulting an optimal detachment and dissociation.

### Preparation and assembly of the bioreactor system (DASbox; for 100–250 mL process scale)

**Timing: 3 days before bioreactor inoculation**

8. Siliconizing of the bioreactor glass vessel
  a. Place the bioreactor glass vessel under the flow hood and repeatedly pipet 1 mL of Sigmacote to the vessel wall (to at least two-thirds of height) and bottom until no liquid is left. This step is necessary to decrease the chance of cells to the vessel to an increased hydrophobicity of the glass. Let the vessel dry for at least 12 h at 20°C–22°C.
  b. Rinse the bioreactor vessel with water in order to remove any Sigmacote residues.
9. Pump calibration (exemplarily shown in Figure 3)
  a. Assemble the base, feed and waste tubing. Important: when the pump head tubing is placed in the pump heads, one tubing end must be long enough to allow placement of tube-attached bottles under the flow hood while being still attached to the bioreactor.
    i. The pump head tubing for the base tubing should have an inner diameter of 0.5 mm. For both the feed and waste tubing, in addition to the 0.5 mm pump head tubing a 1.0 mm pump head tubing must be attached.
  b. Place the pump head tubing of base, feed and waste (starting with the bigger diameter for feed and waste) into the pump heads. Use the DASware software for pump calibration. Prime the lines with pure water.
  c. Weigh empty conical tubes with caps and place the tubing outlets in each conical tube.
  d. Start dispensing at a defined flow rate, which is applied during the cultivation.
  e. Weigh the filled conical with caps and insert the weight of the dispensed water to the software interface.
  f. For feed and waste lines, note the calibration values and repeat steps b–e while using the smaller diameter pump head tubing.

**Figure 3:**
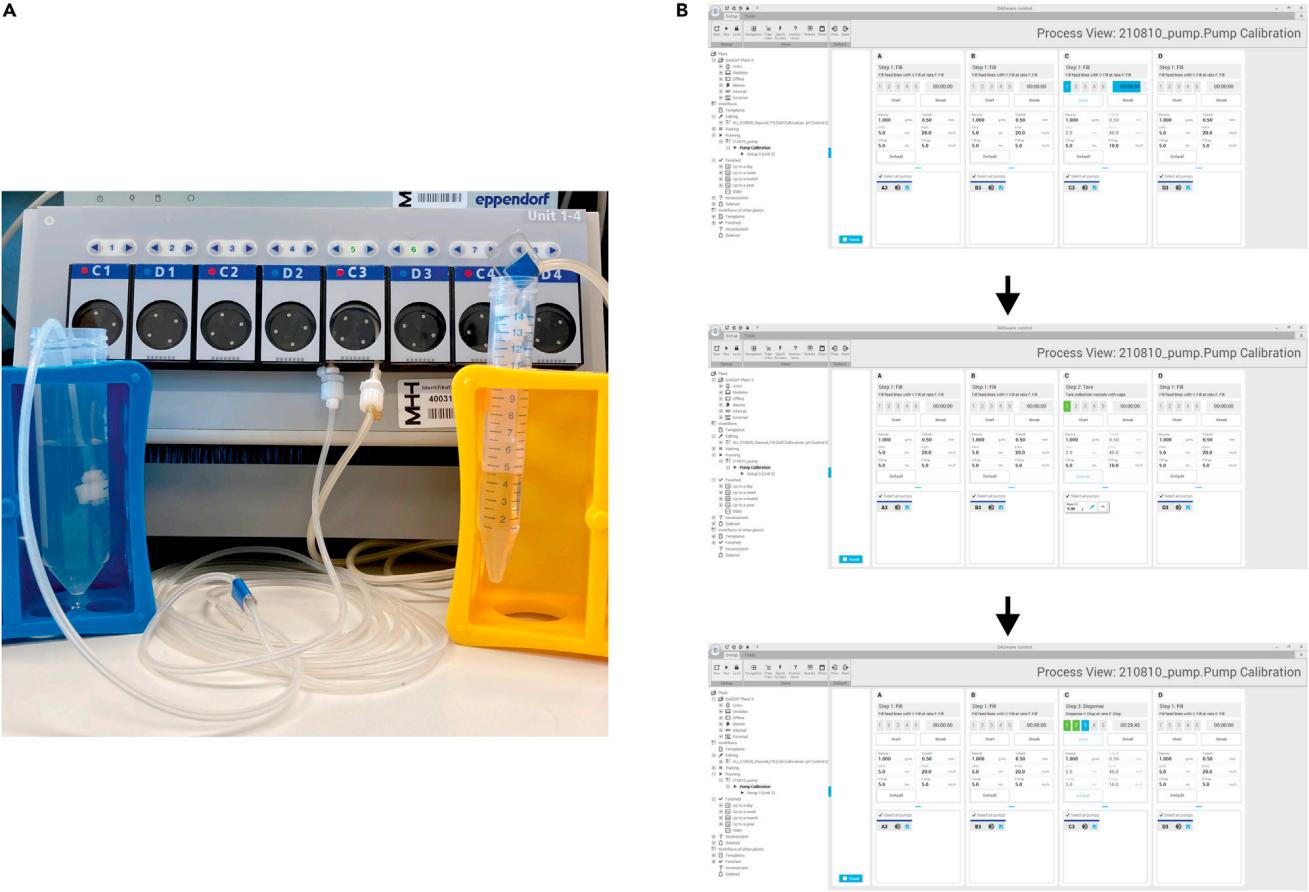
Display of the pump calibration process (A) Exemplary image of a pump calibration process. (B) Exemplary of the software during pump calibration.
10. pH probe calibration
  a. Remove the pH probe from the storage solution, examine the pH probe’s diaphragm for damages and attach the probe to the cable of the bioreactor
  b. Rinse with water, dry with a tissue without rubbing on the diaphragm, and place both the pH probe and the respective temperature probe into the pH 7.00 calibration buffer. It is important to ensure that the diaphragm is fully submerged in the buffer.
  c. After pH and temperature readings have stabilized, calibrate the offset
  d. Repeat steps b and c using pH 4.01 calibration buffer for slope calibration.
11. Assembly of the bioreactor
  a. Assemble the head plate considering the schematic in Figures 4 and 5.

**Figure 4:**
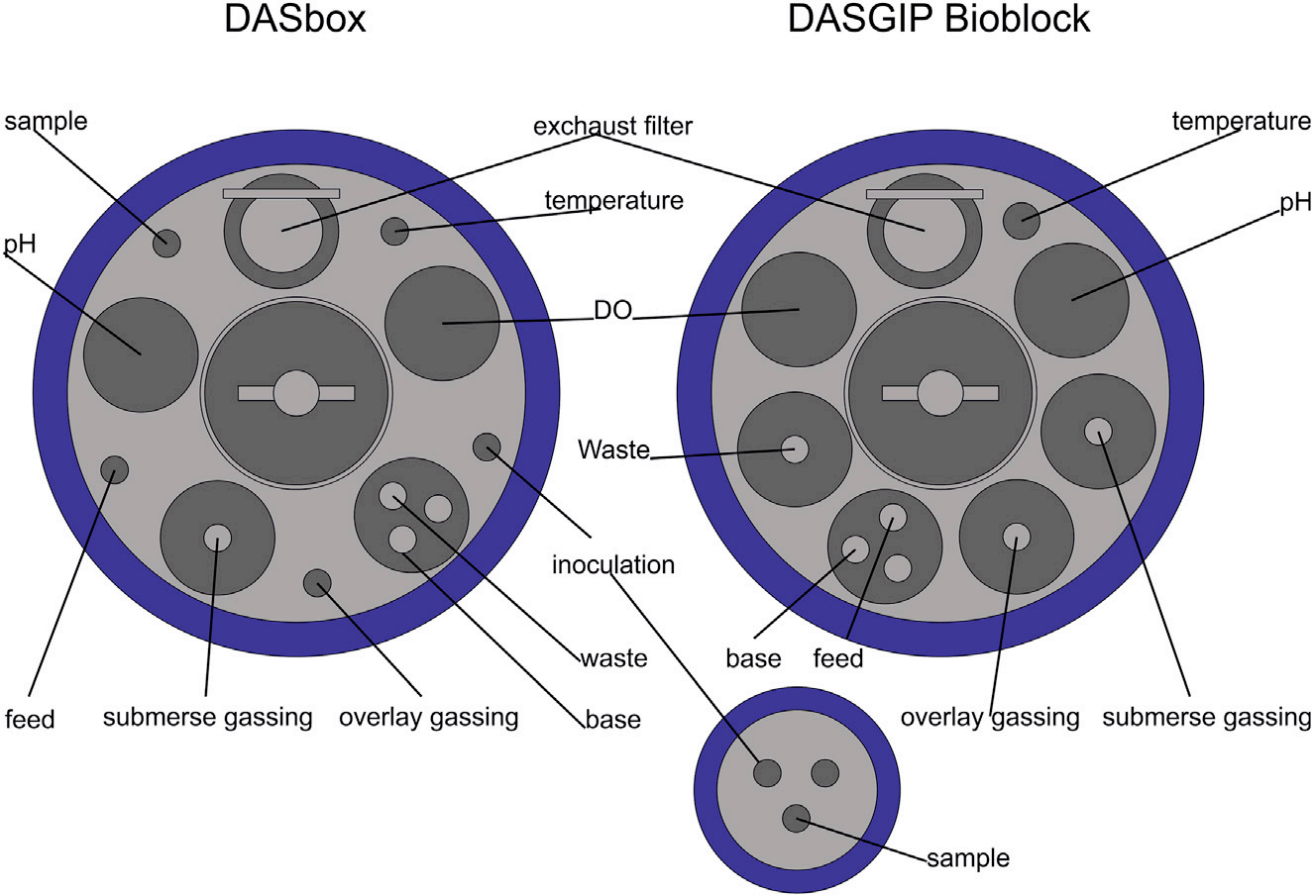
Schematic overview of the headplate arrangement for both DASbox and DASGIP Bioblock bioreactor systems

**Figure 5:**
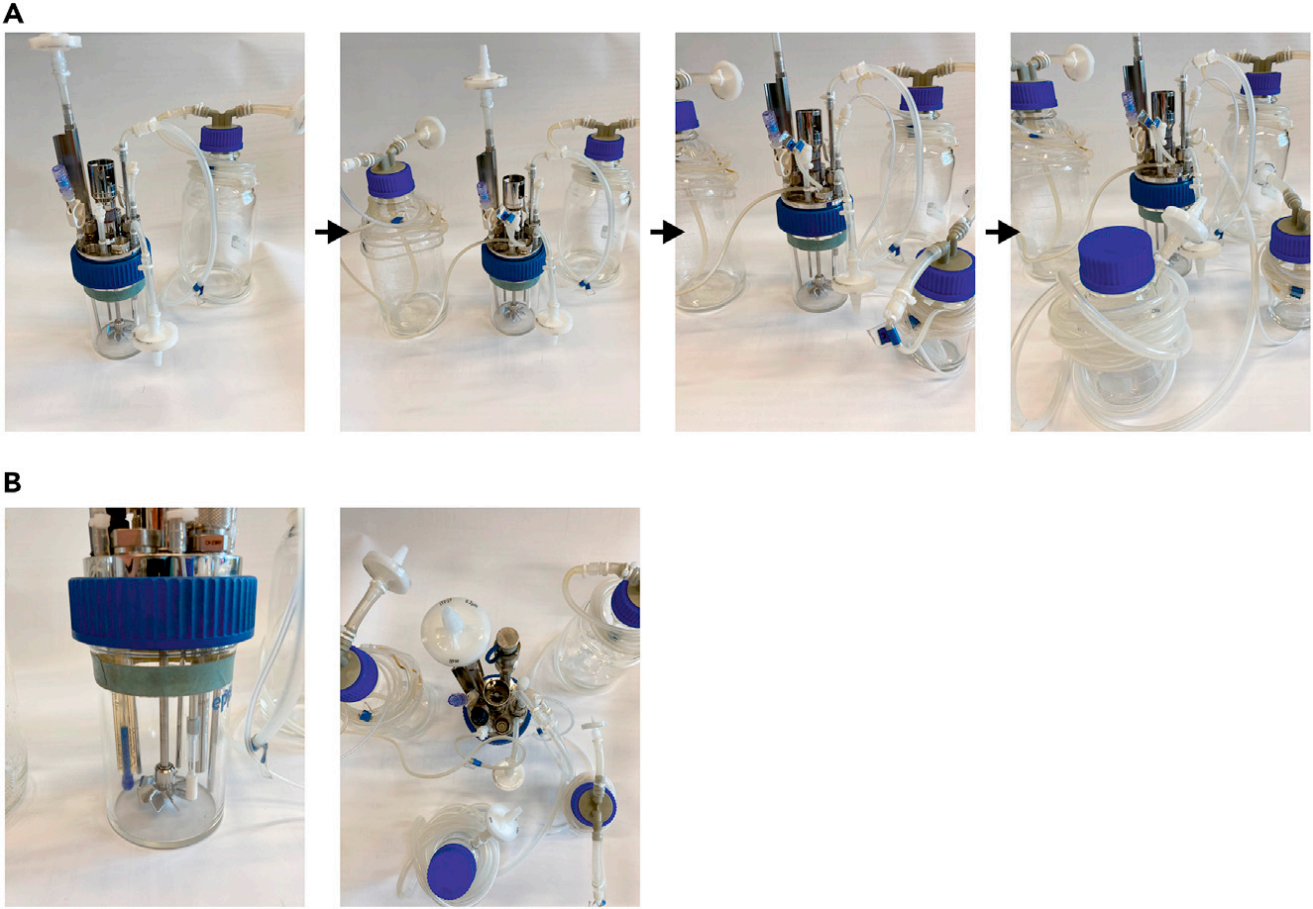
Schematic overview of the assembly of bottles to the DASbox bioreactor (A) Exemplary images of the connection of bottles to the bioreactor. Starting with the waste bottle, followed by feed, base and inoculation. (B) Side and top view of the assembled bioreactor in order to highlight connections.
  b. To enable sampling without process interruption, add a piece of silicone tubing (i.d. 1.0 mm) with a Mohr pinchcock clamp, a female Luer-Lock connector and a Luer-Lock sampling valve to the sample port. Fix connections with cable ties.
  c. Exhaust and inlet air filter:
    i. Add a piece of silicone tubing (i.d. 4.0 mm) with a vent filter to the exit port of the exhaust gas line.
    ii. Assemble a piece of small silicone tubing (i.d. 1.0 mm) with a piece of larger silicone tubing (i.d. 4.0 mm) with the help of a hose reduction piece.
    iii. Attach a vent filter to the larger tube and connect everything to the headspace gassing port (for overlay gassing); fix connections with cable ties.
  d. Attach the impeller (Pitched-Blade Impeller, 8-blade, 60° pitch) at the lowest-possible position of the impeller shaft and tighten with screws in the holding sleeve.
  e. Attach the sintered glass frit with a small piece of tubing to the dip tube and position it directly above the impeller. It will function as a cell retention filter while perfusion.
  f. Attach base-, feed- and waste-tubing with bottles and respective ports at the bioreactor head plate. Note that the longer part of the tubing should always be attached to the bottle (but not to the head plate).
  g. To prepare the inoculation bottle:
    i. attach a silicone tubing (i.d. 4.0 mm; o.d. 6.0 mm) with the hose nozzle at the bottom of the inoculation bottle (Media bottle with two 9 mm hose nozzles).
    ii. Subsequently, add a piece of silicone tubing (i.d. 4.0 mm) with a vent filter to the second hose nozzle at the top of the bottle.
    iii. Attach the Inoculation Bottle to the respective port at the bioreactor.
12. Bioreactor sterilization
  a. Cover the bottom of the bioreactor vessel with pure water in order to allow steam autoclaving and to prevent the pH probes from drying out.
  b. Wrap vent filters with aluminum foil to protect from humidity. Close silicone tubing of the sampling port with the Mohr pinchcock clamp.
  c. Autoclave bioreactor at 120°C for 20 min.
  d. Place the bioreactor vessel into the bioreactor station and connect to the overhead drive, exhaust condenser, temperature sensor, gas supply to the overlay port, and connect the cables of the pH and DO probe.
  e. Remove the water and replace it with 100 mL of sterile PBS with the help of the inoculation bottle. This step should be conducted as soon as possible to prevent the pH probe’s diaphragm from draining.
  f. Place the base tubing in pump B, the feed tubing in pump C and the waste tubing in pump D.
  g. Fill the base bottle with 100 mL of NaHCO_3_ and prime the tubing until one drop of base is dropping into the bioreactor.
13. Dissolved oxygen sensor calibration
  a. Start overlay gassing with 21% O_2_, temperature control at 37°C and stirring at 80 rpm. Let the DO probe polarize for at least 6 h.
  b. The next day, perform one-point slope calibration at 100% DO.

***Optional:*** Additionally apply gassing with pure N_2_ for another 6 h followed by zero point calibration.

**Figure 6:**
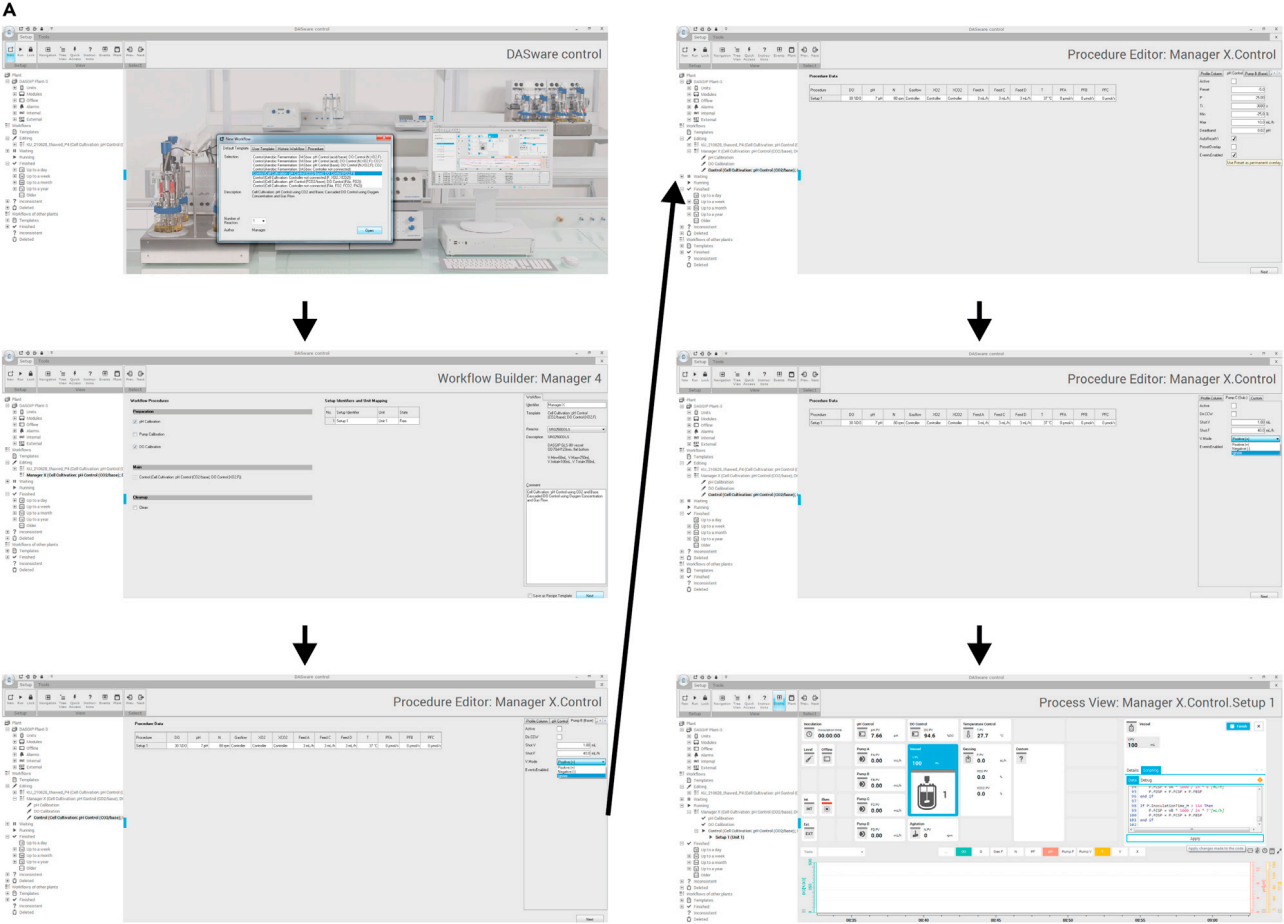
Display of the software set up for bioreactor start

14. Software set-up (shown in Figure 6)
  a. To set up the software first click on “New Workflow”
  b. Under the tab “Default Template” select Control (Cell Cultivation: pH Control (CO2/base); DO Control (XO2, F)). Then select the respective number of reactors to be used and press “open”.
  c. Remove the tick at pH Calibration and DO Calibration, change the name of the process from “Manager X” to your specific process. Subsequently select the bioreactor units to be used; **Optional:** name each unit and press “Next”
  d. In the next window, change the settings by pressing on “Feed C”, the tab “Pump C (Sub.)” and change “V.Mode” to “Ignore”; repeat for “Feed D”. Next press “pH” and the tab ”pH Control” and change the values at “Min” to “−25%” and at “Max” to “10 mL/h”. Subsequently select the tab “Pump B (Base)” and change “V.Mode” to “Ignore”. Finally press “Next”.
  e. Copy the Script by clicking on the individual Unit and press on “Vessel”, select the tab “Scripting”, paste the Script (available under Data S2), adjust the volume to your specific working volume and press “Apply”.

### Preparation and assembly of bioreactor system for larger process scale (DASGIP Bioblock; for 300–1000 mL process scale)

**Timing: 3 days before bioreactor inoculation**

15. The Preparation of the DASGIP Bioblock is analogous to the preparation of the DASbox. Therefore, only changes between the DASGIP Bioblock and the DASbox preparation will be described here.
16. Change to 8. a.
  a. Use 1.5 mL of Sigmacote.
17. Change to 9. a. i.
  a. The tube diameter for feed and waste should be 1.0 mm + 2.0 mm.
18. Change to 12. e.
  a. Fill the bioreactor with 400 mL of PBS after autoclaving.
19. Change to 12. g.
  a. Fill the Base bottle with 400 mL of NaHCO_3_.

### Bioreactor inoculation

**Timing: 1.5 h**

**Figure 7:**
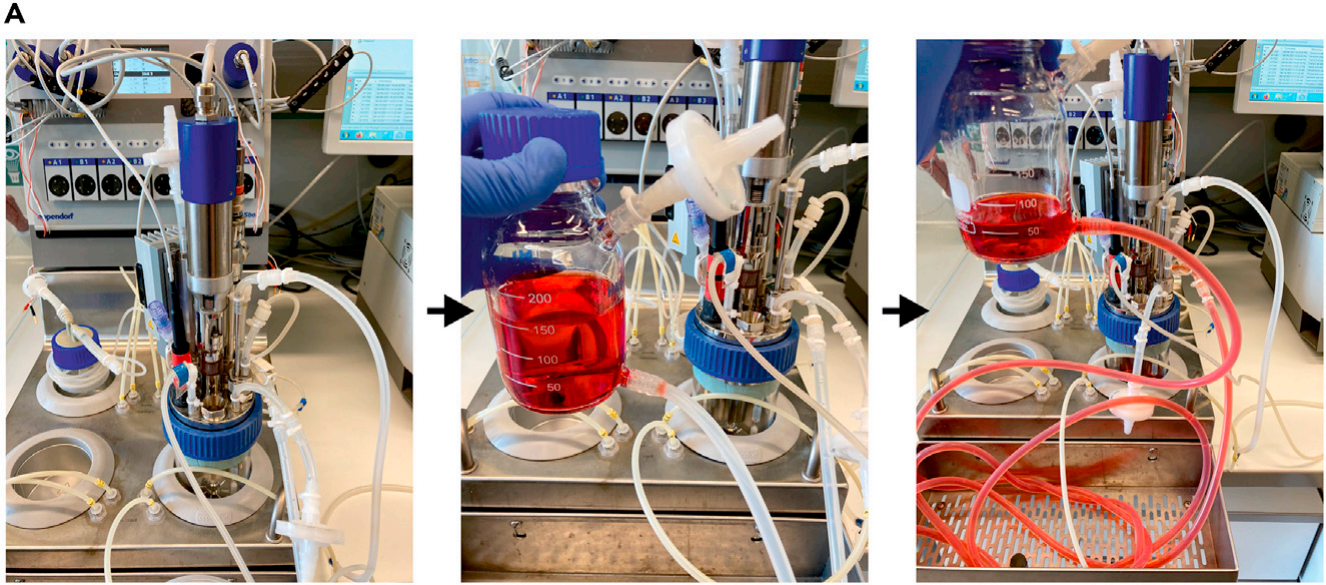
Exemplary images of how an inoculation bottle is used to transfer media and cell suspension into the bioreactor

This section describes how inoculate the bioreactor after the bioreactor was set-up along the description in “Preparation and assembly of the Bioreactor system” (shown in Figure 7)

20. Place the inoculation bottle on a lower level than the bioreactor and transfer the PBS from the bioreactor into the inoculation bottle, by gravity. Tilt the bioreactor to further transfer remaining PBS. Place the inoculation bottle (while being attached to the bioreactor) under the flow hood and discard the PBS.
21. Add 110 mL of fresh E8 suspension medium for bioreactor inoculation to the inoculation bottle, place the inoculation bottle on a higher level than the bioreactor and let it flow by gravity into the bioreactor.
22. Initiate bioreactor agitation, gassing and temperature control.
23. Dissociate the cells from the T-flasks as described in 5; here, the use of a 500 mL centrifugation tube (instead of 50 mL conical tubes) facilitates the process. Resuspend the cell pellet in 100 mL of fresh E8 suspension medium for bioreactor inoculation and determine the cell density of the resulting cell suspension applying the Vi-CELL XR.
24. Calculate the volume of your cell suspension that contains 75 million cells (this cell number is required to achieve the final cell density 0.5 × 10^6^ cells/mL in 150 mL process scale) and transfer the calculated volume into the bioreactor utilizing the inoculation bottle, as described in 21.
25. Add the remaining volume of fresh E8 suspension medium for bioreactor inoculation to fill up to 150 mL to the inoculation bottle and transfer the medium into the bioreactor; this step is required to flush the tubing, thereby avoiding any cell loss in the tubing.
26. Using the software interface, click on Inoculation and Press Start. This step is critical since it defines the (inoculation-) timing for the entire process script. Bioreactor settings are displayed in Table 2.

**Table 2:** Bioreactor settings (exemplarily for a 150 mL DASbox bioreactor)

Stirring	Temperature	Gassing	pH	DO	Perfusion
80 rpm (exact calculation based on Equations 13 + 14) ccw	37°C	0.9 sL/h (reactor volume × 6), 21% O_2_, 5% CO_2_	7.1	40%	see Table 3

**Table 3:** Applied feed rates and feed media for respective culture periods

Culture day	Feed rate in working volumes per day [1/d]	Feed medium
0–1	0	N/A
1–2	1	E8 full feed medium I
2–3	1.5	E8 full feed medium I
3–4	3	E8 full feed medium I
4–5	4	E8 full feed medium II
5–6	6	E8 full feed medium II
6–7	7	E8 full feed medium II
27. As described in “Sampling from the bioreactor”, draw a first process sample at 30 min after inoculation.
  a. The sample can be directly measured via the Vi-CELL XR to test/confirm the desired inoculation cell density aiming at 0.5 × 10^6^ cells/mL; no enzymatic dissociation of the sample is necessary at this point since the cells have not yet attached into aggregates.
28. Fill the feed bottle of the bioreactor with E8 full feed medium I and II; two options can be applied:
  a. If a dedicated fridge is available, the feed bottle can be placed in the fridge at 4°C. Such cooling allows depositing the entire feed medium required for the first 4 days of process duration.
    i. Required volume calculation: process scale multiplied with sum of working volumes during this time span (see Table 2) i.e., 150 × 5.5 = 825 mL of E8 full feed medium I. After 4 days of process duration, additional 2.55 L (at the 150 mL scale) of E8 full feed medium II need to be filled in the feed bottle.
  b. Without the cooling option, only 150 mL of E8 full feed medium I required for the next 24 h of feeding should be deposited in the feed bottle at 20°C–22°C. Subsequently, the feed bottle needs to be refilled on a daily basis based on the applied feed rate (see Table 2).
29. After filling the feed bottle, prime the feed lines.
30. Due to high cell growth, headspace gassing is not sufficient for the whole process duration. Therefore, it needs to be changed to submerse gassing, with a lower gassing rate in order not to damage aggregates by rising bubbles.
  a. After 4 days of process duration. Go to the bioreactor vessel script as described in 14.e. and change the line “Dim FSP as double = VR ∗ 6” to “Dim FSP as double = VR ∗ 1”. Subsequently detach the gassing from the overlay port and attach it to the submerged port in order to supply more oxygen to the culture.

***Note:*** Conduct the process equivalent to steps 20–30 applying the DASGIP Bioblock bioreactor system enabling process scales ranging at 300–1000 mL.

### Sampling from the bioreactor

**Timing: 1.5 h, daily**

This section describes how to draw cell suspension samples from the bioreactor for monitoring cell aggregation and growth kinetics without process interruption. Sampling and sample analysis should be conducted every 24 h (or more frequently on demand).

31. Remove the cap from the bioreactor sample port and spray with 70% Ethanol.
32. Connect a sterile syringe to the valve, create a slight underpressure by pulling the plunger, open the Mohr pinchcock clamp, slowly draw 1 mL of cell suspension into the syringe and close the clamp immediately.
33. After the syringe has been unplugged, discard the cell suspension in order to remove residues that accumulated in the sample line since the last sampling. Subsequently, draw another 2.5 mL of sample volume. Fill the sample into a well of a low attachment 6 well plate. Clean the sample port with 70% ethanol before closing the cap.
34. To assess aggregate morphological appearance and systematic size determination, conduct microscopic images of the hPSC aggregates directly after sampling. Subsequently, transfer 2 mL of the suspension sample into tubes and centrifuge at 300 × g for 3 min. The supernatant can be stored at −20°C before metabolic analysis e.g., to assess glucose, lactate or amino acid contents.
35. For cell counting, wash the obtained cell pellet carefully with 1 mL PBS (avoiding aggregate loss), add 500 μL of Accutase, mix well and incubate for 3 min at 37°C. Centrifuge at 300 × g for 3 min, discard the supernatant, resuspend in 2 mL PBS and determine the cell density with the Vi-CELL XR. Cells can be used for additional analysis such as gene expression patterning, cell cycle assessment or flow cytometry-based monitoring of pluripotency marker expression.

### Flow cytometry-based analysis of pluripotency markers

**Timing: 1 day**

36. Use dissociated cells from the previous step at ∼150000 cells/ well of a 96 well plate.
37. The pluripotency-associated surface markers SSEA-3, SSEA-4 and TRA-1-60 can be triple stained in 50 μL PBS/well applying the following dilutions: 1:25 for SSEA-4 and TRA-1-60, 1:50 (for SSEA-3). Incubate for 12 min at 20°C–22°C.
38. For the intracellular pluripotency- and proliferation-associated markers OCT-3/4, NANOG and KI-67, respectively, treat ∼150000 cells/ well with FIX&PERM® Solution A for 15 min at 20°C–22°C. Conduct triple staining in 50 μL PBS + FIX&PERM® Solution B (1:1 ratio) for 12 min at 20°C–22°C using 1:25 dilutions of each antibody.

### Cleaning of the bioreactor

**Timing: 1 day**

This section describes how to properly clean the bioreactor vessels and tubing before re-usage.

39. For process termination, remove the cell suspension using the inoculation bottle as described in 20. The cells can be further processed as required.
40. Fill the bioreactor with 200 mL of Terg-a-zyme® solution, set the stirring at 150 rpm and incubate at 37°C for at least 12 h.
41. Detach all tubing from the bioreactor, place the detached ends into a 500 mL beaker with ∼500 mL of distilled water and pump backwards into the attached feed-, waste- and base- bottles for 2 h.
42. Detach the tubes from the bottles and place the other end of the tubes into the distilled water-filled beaker to pump in circles for at least 12 h.
43. Rinse all bioreactor ports with Terg-a-zyme® solution. Rinse the cell retention filter thoroughly to avoid future clogging.
44. On the next day, remove the Terg-a-zyme® solution from the bioreactor and replace with distilled water, rinse the ports with distilled water and incubate for 4 h.
45. Remove tubes from the beaker. Continue to pump for 30 min in order to remove liquid residues from tubes.
46. Unscrew DO and pH sensors from the head plate. Place the pH sensor back into the storage solution.
47. Remove the water from the bioreactor vessel and pat dry.

## Expected outcomes

**Figure 8:**
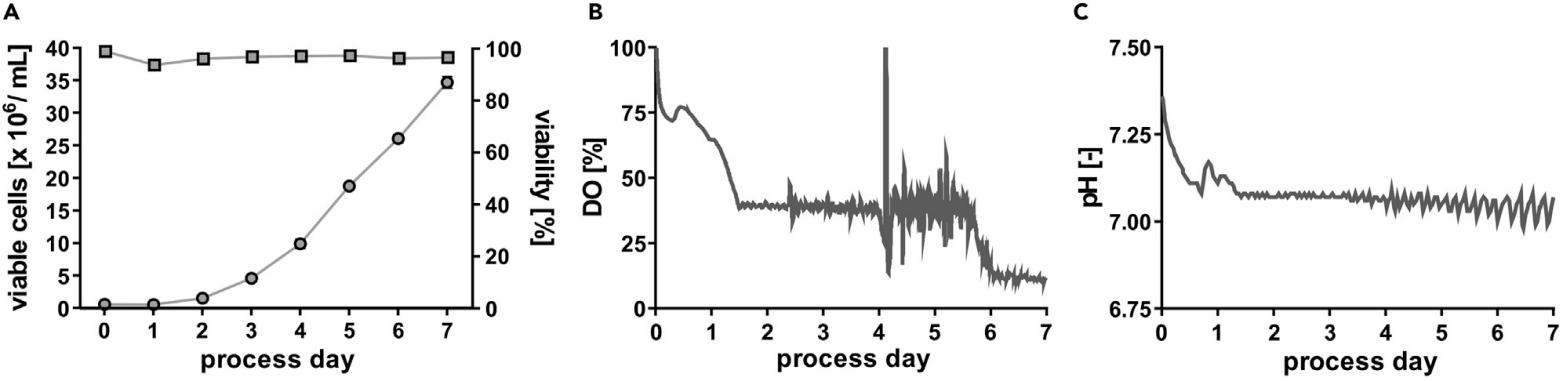
Representative expected results for bioprocesses conducted after this protocol (A) Viable cell density (dots) and viability (squares) for representative processes. (B) Representative online process parameter measurement of dissolved oxygen (DO) value over process time. (C) Representative online process parameter measurement of pH value over process time.

**Figure 9:**
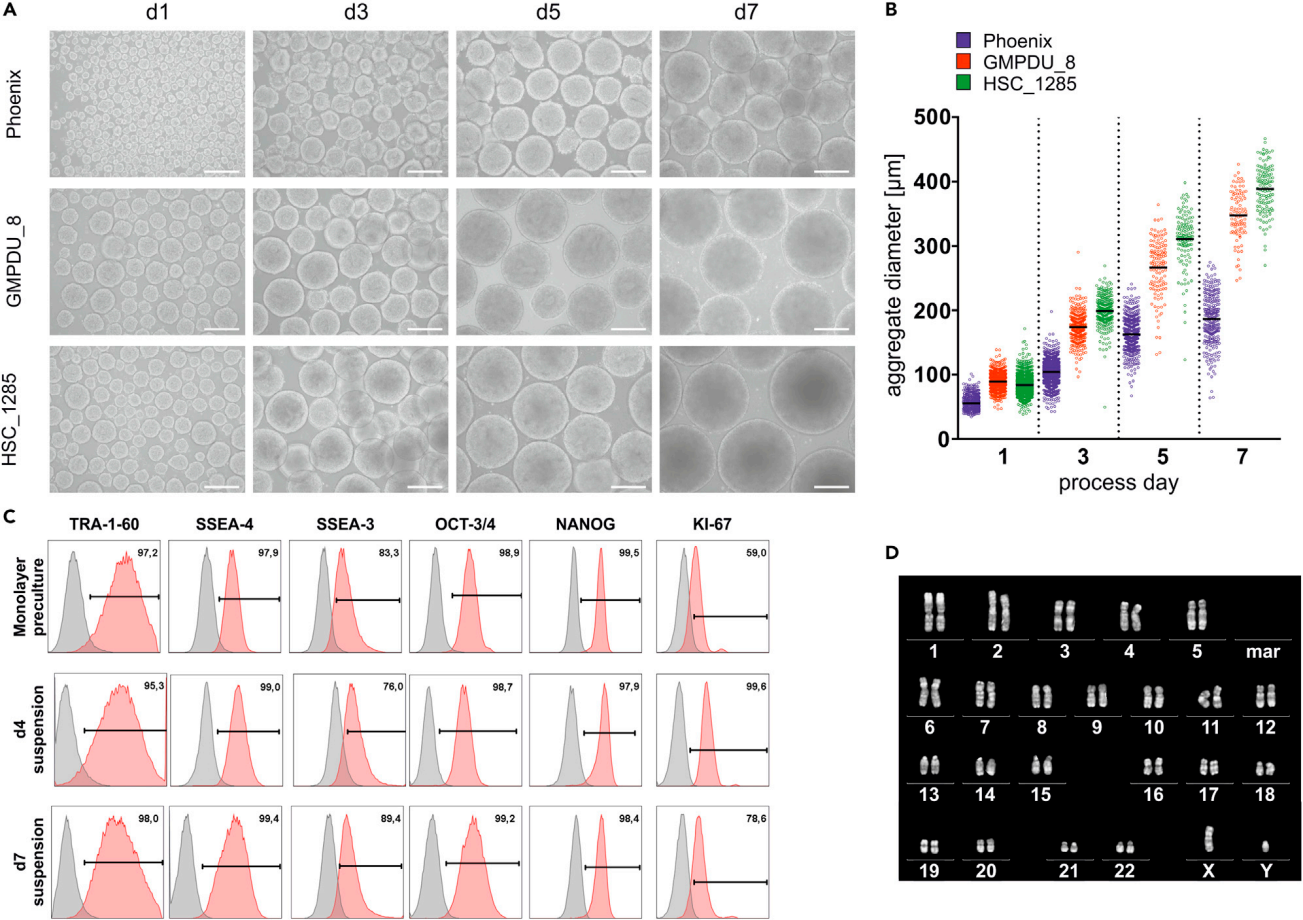
Representative expected results for bioprocesses conducted after this protocol (A) Representative light microscopy pictures of process-derived aggregate samples on days 1, 3, 5 and 7 for 3 different cell lines highlight cell homogeneity within the cultures and the cell line dependent differences in size of formed aggregates(scale bars = 200 μm). (B) Distribution of aggregate diameters over the cultivation time. (C) Representative flow cytometry plots of cells harvested at process endpoint (day 7) showing the pluripotency-associated surface markers TRA-1-60, SSEA-4 and SSEA-3 and transcription factors OCT-3/4 and NANOG as well as the proliferation marker KI-67 (isotype controls shown in gray). (D) Representative karyotype of cells cultured for 7 days under the conditions described here. Figure adapted from (Manstein et al., 2021).

Application of the protocol should enable a cell culture-trained investigator establishing a highly efficient hPSC expansion process characterized by the exponential proliferation of seeded cells between process days 2–5, while further promoting the substantial increase in cell numbers until the suggested process termination on day 7 (potentially followed by re-seeding or initiation of differentiation). The final cell density expected with this protocol is ∼35 × 10^6^ cells/mL on day 7, representing a ∼70-fold expansion of the inoculation density of 0,5 × 10^6^ cells/mL (Figure 8A). Starting with the DO setting of 100%, the triggered DO control (set to 40%) is expected to starts at around 30 h post process inoculation (Figure 8B) and the triggered pH control (set to pH 7.1) shall starts at around 20 h post inoculation (Figure 8C). Due to the high cell densities achieved over time, gassing – which is initially performed via the headspace only - needs to be conducted in a submerged manner (described under 42.) at later stages, which results in a “stroke-like DO pattern” on process day 4, followed by stabilization around 40 % DO thereafter (Figure 7B). Notably, on day ∼5–6, even submerged gassing with our bioreactor setup is insufficient to keep the DO at the envisioned 40 %, therefore dropping down to ∼10 % DO on days 6–7 due to the high cell density-induced oxygen consumption. However, the yielded hPSCs at the proposed process endpoint (day 7) are expected to express high levels of pluripotency-associated markers TRA-1-60, SSEA-4, OCT-3/4, NANOG in >95% of the cell whilst >75% of the cells should express SSEA-3, a marker which we have observed as being most sensitive to improper culture condition (Figure 9C). In addition to the expression of pluripotency-associated markers, cells should display a stable and normal karyotype when assessed at process endpoint (Figure 9D).

Another characteristic of the process is the induction of a homogeneous aggregate suspension, reaching a (somewhat cell line-dependent) mean aggregate diameter of ∼150 μm–350 μm on process day 7 (Figures 9A and 9B). Our observations based on the application of numerous independent cell lines in this protocol suggest that the relative substantial differences in the average aggregate diameter (observed at a given stirring rate on a given process day; Figure 8B) represents cell line-dependent properties of hPSC. On the molecular level, this might be a consequence of the expression level and/ or processing characteristics of E-cadherin or other cell adhesion molecules, as previously suggested (Konze et al., 2014). However, the hPSC aggregates generated by our protocol can be directly applied for lineage-directed differentiation strategies as we have readily demonstrated for cardiomyocytes (Halloin et al., 2019; Manstein et al., 2021). Notably, however, when aiming at lineage-directed differentiation, we do suggest applying hPSC aggregates harvested already on day 3–4 of the process. In contrast to day 7, the cells proliferate exponentially on process day 3–4 and the aggregate diameter is typically below 300 μm, suggesting no/ low diffusion limits to the cells in the core of aggregates. These facts should therefore support both the efficient re-seeding of day 3–4 derived cells (e.g., for “seed train-based” hPSC mass expansion in continuously increasing scales) as well as the robust differentiation of day 3–4 derived aggregates into a cell type of interest, when applying relevant differentiation strategies.

## Quantification and statistical analysis

The below listed equations serve specific requirements in the frame of this protocol as follows.

1. To calculate the cell specific rates that are essential to create the herein used *in silico* model(s), apply the Equations 1, 2, 3, 4, 5, 6, 7, and 8.
2. Use Equations 9, 10, and 11 to compare how well the respective *in silico* model compares to the wet-lab results; the smaller the prediction error, the better the model represents the wet-lab data.
3. Equation 12 describes how the stirring speed must be adapted when aiming at (up)scaling from one to another bioreactor system. However, please note that this calculation is only valid when using impellers of the same geometry across different systems and if the impeller-to-vessel-diameter ratio is constant as well.
4. Use Equations 13 + 14 to calculate and adapt the stirring speed to the applied process volume in the DASbox or the DASGIP Bioblock systems.

The Specific growth rate μ [1/d] can be calculated as

(Equation 1) $$\mu =\left(\frac{X_{t_{n+1}}-X_{t_{n}}}{t_{n+1}-t_{n}}\right)\times \frac{1}{{\bar{X}}_{t_{n+1}}}$$

where *X* is the cell concentration [cells/L] at the given time point *t* and $\bar{X}$ the mean cell concentration [cells/ L] calculated as

(Equation 2) $${\bar{X}}_{t_{n+1}}=\frac{X_{t_{n+1}}-X_{t_{n}}}{\ln\left(X_{t_{n+1}}\right)-\ln\left(X_{t_{n}}\right)}$$

Specific substrate metabolite consumption rates *qsMet* [pmol/ (cell × d)] for the process days without medium change (d0–d1) can be calculated as

(Equation 3) $$\mathrm{qsMe}t_{t_{n+1}}=-\left(\frac{\mathrm{sMe}t_{t_{n+1}}-\mathrm{sMe}t_{t_{n}}}{t_{n+1}-t_{n}}\right)\times \frac{1}{{\bar{X}}_{t_{n+1}}}$$

where s*Met* is the metabolite concentration [pmol/L], *t* the process time point [d].

In the perfused cultures (d1–d7), the specific substrate metabolite consumption rate can be calculated as

(Equation 4) $$\mathrm{qsMe}t_{t_{n+1}}=-\left[\left(\frac{\mathrm{sMe}t_{t_{n+1}}-\mathrm{sMe}t_{t_{n}}}{t_{n+1}-t_{n}}\right)+\frac{F}{V}\left({\bar{\mathrm{sMet}}}_{t_{n+1}}-\mathrm{sMe}t_{f}\right)\right]\times \frac{1}{{\bar{X}}_{t_{n+1}}}$$

where *F* is the flow rate of feed and waste stream [L/day], *V* the culture volume [L], s*Met*_*f*_ the substrate metabolite concentration in the feed stream [pmol/ L] and $\bar{sMet}$ is the mean substrate metabolite concentration in the culture calculated as

(Equation 5) $${\bar{\mathrm{sMet}}}_{t_{n+1}}=\frac{\mathrm{sMe}t_{t_{n+1}}-\mathrm{sMe}t_{t_{n}}}{\ln\left(\mathrm{sMe}t_{t_{n+1}}\right)-\ln\left(\mathrm{sMe}t_{t_{n}}\right)}$$

Specific waste metabolite production rates *qwMet* [pmol/ (cell × d)] for the process days without medium change are calculated accordingly as

(Equation 6) $$\mathrm{qwMe}t_{t_{n+1}}=\left(\frac{\mathrm{wMe}t_{t_{n+1}}-\mathrm{wMe}t_{t_{n}}}{t_{n+1}-t_{n}}\right)\times \frac{1}{{\bar{X}}_{t_{n+1}}}$$

where *wMet* is the waste metabolite concentration [pmol/ L].

In the perfused cultures, the specific waste metabolite production rate is calculated as

(Equation 7) $$\mathrm{qwMe}t_{t_{n+1}}=\left[\left(\frac{\mathrm{wMe}t_{t_{n+1}}-\mathrm{wMe}t_{t_{n}}}{t_{n+1}-t_{n}}\right)+\frac{F}{V}{\bar{\mathrm{wMet}}}_{t_{n+1}}\right]\times \frac{1}{{\bar{X}}_{t_{n+1}}}$$

where $\bar{wMet}$ is the mean waste metabolite concentration in the culture calculated as

(Equation 8) $${\bar{\mathrm{wMet}}}_{t_{n+1}}=\frac{\mathrm{wMe}t_{t_{n+1}}-\mathrm{wMe}t_{t_{n}}}{\ln\left(\mathrm{wMe}t_{t_{n+1}}\right)-\ln\left(\mathrm{wMe}t_{t_{n}}\right)}$$

To calculate the prediction error of a modeling approach, first the relative error for each individual data point pair (*e*_*ri*_) needs to be calculated as follows, where *x*_*wl*_ are wet-lab and *x*_*m*_ are modeled data points, with $x_{wl\;max}$ as the maximal wet-lab data point:

(Equation 9) $$e_{r_{i}}=\frac{\left|x_{\mathrm{wl}}-x_{m}\right|}{x_{\mathrm{wl max}}}\times 100$$

The average error ${\bar{e}}_{r}$ provides the value on how well the model represents the wet-lab data. The average error and the standard deviation for the average error $SD_{{\bar{e}}_{r}}$ for each data set can be calculated as follows:

(Equation 10) $${\bar{e}}_{r}=\frac{\sum_{i=1}^{n}e_{r_{i}}}{n}$$

(Equation 11) $$SD_{{\bar{e}}_{r}}=\sqrt{\frac{1}{n-1}\times \overset{n}{\underset{i=1}{\sum }}{\left(e_{r_{i}}-{\bar{e}}_{r}\right)}^{2}}$$

The impeller rotational speed *N* [rps] of the new vessel *o*, can be calculated with the impeller diameter *D* [m], the impeller rotational speed and the vessel liquid volume *V* [m^3^] of the new and the previously used vessel *i*:

(Equation 12) $$N_{o}=\sqrt[{3}]{N_{i}^{3}\times \frac{V_{o}}{V_{i}}\times \frac{D_{i}^{5}}{D_{o}^{5}}}$$

For calculation of the impeller rotational speed at a given liquid volume in the DASbox the following equation can be used:

(Equation 13) N=150.5×V13

For calculation of the impeller rotational speed at a given liquid volume in the DASGip Bioblock the following equation can be used:

(Equation 14) N=76.5×V13

## Limitations

As noted above, it is expected that the here described expansion strategy should yield similar cell densities for other hPSC lines that are of interest to any other laboratory. However, it cannot be excluded that the aggregation of individual cell lines is either poor (thus limiting/precluding the proposed suspension culture approach) or, in contrast, results in the excessive uncontrolled clumping of hPSCs, limiting proliferation and pluripotency. Moreover, cell line specific metabolic properties might differ from the cell lines tested by us, requiring the adjustment of cell specific metabolic rates. Those can be retrospectively adapted and integrated into our *in silico* modeling approach (according to Equations 3, 4, 5, 6, 7, and 8) after conducting preliminary wet lab experiments (to generate the required database) applying the culture strategies described here.

As another limitation, it should be noted that the model described here is only validated for the key variables (i.e., glucose-, glutamine- and lactate- levels, osmolality and aggregate size) investigated by us. Thus, future adaptation e.g., to media properties such as temperature-induced decay of FGF-2 levels may be required, respectively.

Also of note: The upscaling strategy described here is only valid when 1) applying an impeller design representing the same (or highly equivalent) geometry and when 2) the impeller-to-vessel diameter ratio remains essentially constant.

As a final limitation, our *in silico* process model is only validated for modeling the growth kinetics of hPSCs at the pluripotent state but not yet adapted to modeling the complex transition or pluripotent hPSCs along their differentiation into specific lineages.

## Troubleshooting

### Problem 1

The monolayer culture is too dense on the passaging day i.e., exceeding 80% confluence (step 5). In addition, the media is bright yellow and excessive dead cells and debris are microscopically observable in the supernatant.

### Potential solution

Improper cell numbers (i.e., too high) were seeded. Ensure proper counting and dilution of your cells before seeding. Eventually, reduce the cell number used for seeding as this adaptation may be required for your line. In addition, keep track of the splitting rhythm: It should always be maintained for three days. Eventually check your cell line for normal karyotype or even test the genomic integrity at higher resolution applying respective technologies (Baker et al., 2016) to exclude undesired cell transformation.

### Problem 2

The monolayer culture is below ∼60% of confluence and only small cell colonies are observed after the suggested three days of passage duration (step 5).

### Potential solution

This suggests seeding of low cell numbers: Ensure proper counting and dilution of your cells before seeding. Eventually increase the cell number used for seeding as this adaptation maybe required for your hPSC line. Remember to add RI when passaging hPSCs. In addition, check the quality of the growth factors i.e., FGF-2, TGF-ß or Insulin (Massai et al., 2017) in the E8 adherent medium and eventually use fresh medium/ growth factors. Eventually try alternative suppliers of critical media components, in particular of the growth factors highlighted above. Keep track of the splitting rhythm to be maintained strictly for three days (see problem 1).

### Problem 3

The monolayer culture appears differentiated, which should be validated by microscopic assessment (Zweigerdt et al., 2011) or by flow cytometry (step-by-step method details, steps 36–38 quantification and statistical analysis) on the day of passaging (i.e., on day 3, step 5).

### Potential solution

Critically check the quality of your growth factors stocks (origin/supplier, shelf-life, concentration) used to generate the E8 adherent medium and eventually use fresh medium or ensure growth factor replacement; do not conduct repeated freeze/thaw cycles of your growth factor stocks.

In addition, keep track of the feeding and splitting rhythm, do not prolong these beyond three days, and avoid exceeding 80% of cell confluence (see problems 1, 2 and Figure 2D). Regularly check your culture for the level of pluripotency markers expression by flow cytometry (step-by-step method details, quantification and statistical analysis, steps 36–38).

### Problem 4

24 h after bioprocess inoculation, cells did not form any or very little aggregates (after step 29).

### Potential solution

Ensure proper quality of your 2D culture used for process inoculation by considering problems 1, 2, and 3 above. Moreover, avoid extensive incubation time with Accutase (i.e., do not exceed 3 min; see step 5) when generating single cells for cell passaging in 2D and for generating the cell suspension for bioreactor inoculation.

Reevaluate the culture media to ensure proper supplementation of RI and Pluronic™ F-68 in the E8 suspension medium (for bioreactor inoculation). In addition, verify that the stirring speed was set according to step 26 and Equations 12, 13, and 14.

### Problem 5

Cell aggregates remain smaller than the scope exemplified in Figure 9B throughout the process.

### Potential solution

Reduce the stirring speed in adaptation to your hPSC line but keeping in mind that stirring speed reduction may also increase the heterogeneity of the aggregate diameter distribution; do not fall below the minimal stirring speed of 50 rpm at 150 mL process volume with respect to the DASbox system. Note that the minimum speeds for other culture volumes can be calculated according to Equations 12, 13, and 14.

### Problem 6

Cell aggregates become large than the scope exemplified in Figure 9B throughout the process

### Potential solution

Increase the stirring speed according to your specific culture process and hPSC line in hand, but do not exceed a maximum speed of 120 rpm at 150 mL process scale for the DASbox system, as exemplified for this protocol. Based on our experience, higher stirring speed may harm your cells and will reduce cell viability and yield (Manstein et al., 2021). The maximum stirring speeds for other culture volumes (and potentially bioreactor systems) can be calculated according to Equations 12 or 13. Ensure the supplementation of Pluronic™ F-68 to your E8 suspension medium (for bioreactor inoculation), E8 full feed medium I and II as this is of key importance to protect cells from shear-induced damage.

### Problem 7

The cell density at 24 h after bioreactor inoculation is (substantially) below 0.4 × 10^6^ cells/mL (i.e., >20 % below the intended seeding density of 0.5 × 10^6^ cells/mL; see process step 24).

### Potential solution

Ensure proper attention to the hPSC culture used for inoculation by referring to problems 1, 2, and 3 above. Reevaluate the E8 suspension medium (for bioreactor inoculation) in particular for the proper concentration and quality of RI and Pluronic™ F-68.

### Problem 8

Extensive cell clumping eventually plugging narrow gaps in the bioreactor setup such as gaps between the pH or DO probe and the vessel wall. The formation of extensive cell clumps or even cell layers attaching to the bottom of the vessel or other parts inside the bioreactor (i.e., probes, sampling ports, etc.) is observed. The (localized) accumulation and extensive clumping of cells may entirely deplete the cell suspension and subsequently disrupt the bioprocess (after step 29).

### Potential solution

Reevaluate that proper coating of the vessel was performed according to step 8 of this protocol.

Make sure that during bioreactor assembly proper gaps between the respective probe(s) and the vessel wall remain; ensure to place the bioreactor lid properly, which is a prerequisite to avoid any direct attachment of any installations inside the bioreactor and the vessel wall.

### Problem 9

Application of an alternative bioreactor system and/or impeller type (compared to the model described here, step 8 and following) may result in improper cell aggregation, poor cell yields etc.

### Potential solution

Generally, performing the protocol in other bioreactor systems will require systematic testing and modifications. Using another impeller design, it should be noted as a “rule of thumb” that the application of a lower number of blades (an 8-blade pitched impeller is used here; see key resources table), will typically require an increase in the stirring speed compared to this protocol and vice versa (higher number of blades requesting lower stirring speed).

### Problem 10

The pH/DO sensor providing no/ or (apparently) wrong signals (following step 14).

### Potential solution

Check that the connection of cables and probes is dry and well connected.

Ensure that the time when the pH sensor is not submerged in the liquid is minimized; extended time periods can lead to damage of the pH sensor requiring replacement. In this case, the sensor needs to be replaced, recalibrated and sterilized before use.

The electrolyte of the DO sensor may be depleted after autoclaving. In this case, replacement of the membrane and the electrolyte is required following manufacturer’s instructions; ensure autoclaving of the reactor after fixing any probe-related or other issues.

### Problem 11

Improper media prime feed or base lines due to pump failure (step 29).

### Potential solution

Check that all luer connections are tight and no tubing or luer connector is broken. If no connection issues are observed, it might be helpful to use a pipette boy for applying some external pressure to the vent filter of the respective bottle, thereby pushing the liquid into the reactor; this handling requires temporary removal of the pump head to allow liquid flow; ensure subsequent reintroduction of the pump head, respectively.

### Problem 12

Increasing bioreactor liquid volumes are accumulating during perfusion mode (following step 29).

### Potential solution

Check the calibration of pumps B, C, D by using the DASWare Software, clicking on the respective pump, choosing the tab “Adjust” and checking the value at “F.Cal”. The value should be at ∼100 1/mL for a tube diameter of 0.5 mm and at ∼33 1/mL for a tube diameter of 1.0 mm. Compare the values for all 3 pumps. In case of major differences between the calibrations, adjust the values to their mean.

Check that all luer connections are tight and no tubing or luer connector is broken.

Check whether the retention filter is blocked. This becomes visible when small bubbles rise from the waste port, suggesting that the retention filter needs to be changed. In this case, stop temperature, pH and DO controls, agitation and pumps in the DASWare software, disconnect the overhead drive, exhaust condenser, temperature sensor, gas supply, and disconnect the cables to the pH and DO probe. Take the tubings out of the pump heads and place the whole bioreactor under the flow hood. Take another retention filter, that was autoclaved separately and replace the blocked filter with the new one by opening the bioreactor with the help of forceps. Subsequently, close the bioreactor, place it back into the station, reconnect all respective lines and restart temperature, pH and DO controls, agitation and pumps.

## Resource availability

### Lead contact

Further information and requests for resources and reagents should be directed to and will be fulfilled by the Lead Contact, Robert Zweigerdt (zweigerdt.robert@mh-hannover.de), or technical contact, Felix Manstein (Manstein.felix@mh-hannover.de).

### Materials availability

This study did not generate new unique reagents.

## Data Availability

The published article by Manstein et al. includes all datasets generated or analyzed during this study. All codes necessary to reproduce the data are provided in this protocol.

## Supplemental information

Data S1. Berkeley Madonna model code for high density expansion of hPSCs, related to step 1 of the before you begin section

Data S2. DASware script for high density expansion of hPSCs, related to step 14 e
