# Supplementary material for: Process control and in silico modeling strategies for enabling high density culture of human pluripotent stem cells in stirred tank bioreactors
Source: STAR Protoc. 2021 Dec 9;2(4):100988. doi: 10.1016/j.xpro.2021.100988 (PMC8666714; doi:10.1016/j.xpro.2021.100988)
Supplement: Data S1. Berkeley Madonna model code for high density expansion of hPSCs, related to step 1 of the before you begin section [file mmc1.pdf]

```
METHOD RK4

STARTTIME = 0
STOPTIME=7

DT = 0.02

{start parameters}

X0 = 500000000             {cells/L; starting cell concentration}
Glc0 = 17.5          {mmol/L; starting glucose concentration}
Lac0 = 0             {mmol/L; starting lactate concentration}
Gln0 = 2.5           {mmol/L; starting glutamine concentration}
V0 = 0.15            {L; used reactor volume}
Osmo0 = 318          {mOsm/kg; starting osmolality}
Agg0 = 17            {µm, size of a single cell}
SFLac = 0            {mM; lactate concentration in the feed medium}

YXG = um/(qGlc*0.75)
YXL = um/(qLac*0.75)
YXGln = um/qGln
mG = qGlc*0.15
mL = qLac*0.15
qGlc = 1.474e-8            {mmol/Zelle*d; cell specific glucose consumption rate}
qGln = 1.856e-9            {mmol/Zelle*d; cell specific glutamine consumption rate }
qLac = 2.37e-8       {mmol/Zelle*d; cell specific lactate production rate}
um = 1.35            {1/d; maximal cell specific growth rate}
Kglc = 1.5           {mmol; Monod constant for glucose}
Kgln = 0.1           {mmol; Monod constant for glutamine}
Klac = 65            {mmol; Monod constant for lactate}
Kagg = 350/2         {µm; Monod-like constant for aggregate size}
KOsmo = 500          {mOsm; Monod-like constant for osmolality}
am = 0.25            {aggregate growth factor}
ad1 = 0.95           {aggregate formation factor}

{initiators}

init X = X0
init Glc = Glc0
init Lac = Lac0
init Gln = Gln0
init Glc2 = Glc0
init Lac2 = Lac0
init Agg = Agg0
init Osmo = Osmo0

{balances}
```

```
d/dt(X) = u * X-1.9*u*o*X
d/dt(Glc) = -u/YXG * X - F / V0 * ( Glc - SFGlc ) - mG * X
d/dt(Lac) = u/YXL *  X - F / V0 * ( Lac - SFLac ) + mL * X
d/dt(Gln) = -u/YXGln *  X - F / V0 * ( Gln - SFGln )
d/dt(Glc2) =- F / V0 * (Glc2 - SFGlc )
d/dt(Lac2) =  u/YXL *  X  + mL * X
Base = 0.1873 * Lac2 -1.5767
d/dt(Agg) = ag * Agg * 1.7

{kinetics}

u =  um * (Glc/(Kglc + (1+ (Lac/Klac)) * Glc)) * (Gln/(Kgln+Gln)) *
(Kagg^9/((Agg/2)^9+Kagg^9)) * (KOsmo^9/(Osmo^9+KOsmo^9))
d/dt(Osmo) =if time <= 1.5 then 0 else  0.06 * ( F / V0 * SFGlc * 9.25 - (u/YXL *  X  + mL * X) *
3.5 -  (u/YXG * X - F / V0 * ( Glc - SFGlc ) - mG * X )) / (F/V0)  - F / V0 * (Glc2 - SFGlc )
ag = h * (((am * u))-0.03)   + d * ad1

{Feeding}
F = a*(F2a+F3a+F4a+F5a+F6a+F7a)              {L/d}
a = 1
b= if TIME >= 2 then 0 else 1
d= if TIME >= 1 then 0 else 1
h= if TIME <= 1 then 0 else 1
m= if TIME >= 6 then 0 else 1
n= if TIME <= 6 then 0 else 1
o= if TIME >= 0.5 then 0 else 1
p= if TIME <= 0.5 then 0 else 1

SFGlc = a*( SFGlcd1a+ SFGlcd2a+ SFGlcd3a+ SFGlcd4a+ SFGlcd5a+ SFGlcd6a)                {mM;
glucose concentration in the feed medium}
SFGln = a*( SFGlnd1a+ SFGlnd2a+ SFGlnd3a+ SFGlnd4a+ SFGlnd5a+ SFGlnd6a)               {mM;
glutamine concentration in the feed medium }

tf2 = 1
tf3 = 2
tf4 = 3
tf5 = 4
tf6 = 5
tf7 = 6
tf8 = 7

F2 = V0 * 1               {L}
F3 = V0 * 1.5             {L}
F4 = V0 * 3               {L}
F5 = V0 * 4               {L}
F6 = V0 * 6               {L}
F7 = V0 * 7               {L}
```

```
F2a = if time <= tf2 then 0 else if time >= tf3 then 0 else F2
F3a = if time <= tf3 then 0 else if time >= tf4 then 0 else F3
F4a = if time <= tf4 then 0 else if time >= tf5 then 0 else F4
F5a = if time <= tf5 then 0 else if time >= tf6 then 0 else F5
F6a = if time <= tf6 then 0 else if time >= tf7 then 0 else F6
F7a = if time <= tf7 then 0 else F7

SFGlcd1 = 34.15        {mM}
SFGlcd2 = 34.15        {mM}
SFGlcd3 = 34.15        {mM}
SFGlcd4 = 42.5                 {mM}
SFGlcd5 =42.5          {mM}
SFGlcd6 = 42.5                 {mM}

SFGlcd1a = if time <= tf2 then 0 else if time >= tf3 then 0 else SFGlcd1
SFGlcd2a = if time <= tf3 then 0 else if time >= tf4 then 0 else SFGlcd2
SFGlcd3a = if time <= tf4 then 0 else if time >= tf5 then 0 else SFGlcd3
SFGlcd4a = if time <= tf5 then 0 else if time >= tf6 then 0 else SFGlcd4
SFGlcd5a = if time <= tf6 then 0 else if time >= tf7 then 0 else SFGlcd5
SFGlcd6a = if time <= tf7 then 0 else SFGlcd6

SFGlnd1 = 4.5          {mM}
SFGlnd2 = 4.5          {mM}
SFGlnd3 = 4.5          {mM}
SFGlnd4 = 5            {mM}
SFGlnd5 = 5            {mM}
SFGlnd6 = 5            {mM}

SFGlnd1a = if time <= tf2 then 0 else if time >= tf3 then 0 else SFGlnd1
SFGlnd2a = if time <= tf3 then 0 else if time >= tf4 then 0 else SFGlnd2
SFGlnd3a = if time <= tf4 then 0 else if time >= tf5 then 0 else SFGlnd3
SFGlnd4a = if time <= tf5 then 0 else if time >= tf6 then 0 else SFGlnd4
SFGlnd5a = if time <= tf6 then 0 else if time >= tf7 then 0 else SFGlnd5
SFGlnd6a = if time <= tf7 then 0 else SFGlnd6

{limits}

limit Glc >= 0
limit Gln >= 0
limit Lac >= 0
limit Base >= 0
```

```
Dim VR as double = 0.15 '[L]
Dim FSP as double = VR * 6
Dim DOSP as double = 40
Dim PHSP as double = 7.1
Dim FBSP as double
Dim FCSP as double = VR * 1000 / 24 '[mL/h]
Dim FDSP as double = FCSP + FBSP
Dim TSP as double = 37 '[°C]
Dim NSP as double = 150.5 * (VR ^ (1/3)) '[rpm]
Dim PumpBActive as boolean
Dim PumpCActive as boolean
Dim PumpDActive as boolean
Dim TWait as double = 24 ' Wait before pump start [h]
Dim InoculationTime_H as double

Dim PHP as double = 10
Dim PHTi as double = 3600 '[s]
Dim PHDeadband as double = 0.02

With P
        .FSP = FSP
        .DOSP = DOSP
        .PHSP = PHSP
        .FCSP = FCSP
        .FDSP = FDSP
        .TSP = TSP
        .NSP = NSP
        .DirCCW = true
        .PHP = PHP
        .PHTi = PHTi
        .PHDeadband = PHDeadband
end with

If P.InoculationTime_H > 2 then
        if P.PumpDActive = False Then
                If P.InoculationTime_H > TWait then
                        P.PumpCActive = True
                        P.PumpDActive = True
                        P.LogMessage("Start of perfusion")
                end if
        end if
If P.DOActive = False Then
                If P.DOPV < 40 Then
                        P.DOActive = True
                        P.LogMessage("Start of DO control")
                end if
        end if
```

```
        If P.PHActive = False Then
                If P.PHPV < 7.1 Then
                        P.PHActive = True
                        P.PumpBActive = True
                        P.LogMessage("Start of pH control")
                end if
        end if
end if

If P.InoculationTime_H > 48 Then
        P.FCSP = VR * 1000 / 24 * 1.5'[mL/h]
        P.FDSP = P.FCSP + P.FBSP
end if

If P.InoculationTime_H > 72 Then
        P.FCSP = VR * 1000 / 24 * 3'[mL/h]
        P.FDSP = P.FCSP + P.FBSP
end if

If P.InoculationTime_H > 96 Then
        P.FCSP = VR * 1000 / 24 * 4'[mL/h]
        P.FDSP = P.FCSP + P.FBSP
end if

If P.InoculationTime_H > 120 Then
        P.FCSP = VR * 1000 / 24 * 6'[mL/h]
        P.FDSP = P.FCSP + P.FBSP
end if

If P.InoculationTime_H > 144 Then
        P.FCSP = VR * 1000 / 24 * 7'[mL/h]
        P.FDSP = P.FCSP + P.FBSP
end if
```
