# Supplementary material for: Process control and in silico modeling strategies for enabling high density culture of human pluripotent stem cells in stirred tank bioreactors
Source: STAR Protoc. 2021 Dec 9;2(4):100988. doi: 10.1016/j.xpro.2021.100988 (PMC8666714; doi:10.1016/j.xpro.2021.100988)
Supplement: Data S2. DASware script for high density expansion of hPSCs, related to step 14 e [file mmc2.pdf]

```
Dim VR as double = 0.15 '[L]
Dim FSP as double = VR * 6
Dim DOSP as double = 40
Dim PHSP as double = 7.1
Dim FBSP as double
Dim FCSP as double = VR * 1000 / 24 '[mL/h]
Dim FDSP as double = FCSP + FBSP
Dim TSP as double = 37 '[°C]
Dim NSP as double = 150.5 * (VR ^ (1/3)) '[rpm]
Dim PumpBActive as boolean
Dim PumpCActive as boolean
Dim PumpDActive as boolean
Dim TWait as double = 24 ' Wait before pump start [h]
Dim InoculationTime_H as double

Dim PHP as double = 10
Dim PHTi as double = 3600 '[s]
Dim PHDeadband as double = 0.02

With P
	.FSP = FSP
	.DOSP = DOSP
	.PHSP = PHSP
	.FCSP = FCSP
	.FDSP = FDSP
	.TSP = TSP
	.NSP = NSP
	.DirCCW = true
	.PHP = PHP
	.PHTi = PHTi
	.PHDeadband = PHDeadband
end with

If P.InoculationTime_H > 2 then
	if P.PumpDActive = False Then
		If P.InoculationTime_H > TWait then
			P.PumpCActive = True
			P.PumpDActive = True
			P.LogMessage("Start of perfusion")
		end if
	end if
If P.DOActive = False Then
		If P.DOPV < 40 Then
			P.DOActive = True
			P.LogMessage("Start of DO control")
		end if
	end if
```

```
        If P.PHActive = False Then
                If P.PHPV < 7.1 Then
                        P.PHActive = True
                        P.PumpBActive = True
                        P.LogMessage("Start of pH control")
                end if
        end if
end if

If P.InoculationTime_H > 48 Then
        P.FCSP = VR * 1000 / 24 * 1.5'[mL/h]
        P.FDSP = P.FCSP + P.FBSP
end if

If P.InoculationTime_H > 72 Then
        P.FCSP = VR * 1000 / 24 * 3'[mL/h]
        P.FDSP = P.FCSP + P.FBSP
end if

If P.InoculationTime_H > 96 Then
        P.FCSP = VR * 1000 / 24 * 4'[mL/h]
        P.FDSP = P.FCSP + P.FBSP
end if

If P.InoculationTime_H > 120 Then
        P.FCSP = VR * 1000 / 24 * 6'[mL/h]
        P.FDSP = P.FCSP + P.FBSP
end if

If P.InoculationTime_H > 144 Then
        P.FCSP = VR * 1000 / 24 * 7'[mL/h]
        P.FDSP = P.FCSP + P.FBSP
end if
```
